# Supplementary material for: Plasma proteomic associations with genetics and health in the UK Biobank
Source: Nature. 2023 Oct 4;622(7982):329–38. doi: 10.1038/s41586-023-06592-6 (PMC10567551; doi:10.1038/s41586-023-06592-6)
Supplement: Supplementary file 1 — Supplementary Text, Supplementary Figs. 1–14, Supplementary Tables 31–33 and Supplementary References. [file 41586_2023_6592_MOESM1_ESM.pdf]

nature portfolio

https://doi.org/10.1038/s41586-023-06592-6

Supplementary information

Plasma proteomic associations with genetics and health in the UK Biobank

In the format provided by the authors and unedited

Supplementary Information

Table of Contents

Overview of the UKB-PPP study 2

UK Biobank sample selection for UKB-PPP 2

UKB sample handling 4

Plasma profiling using the Olink technology 4

NPX calculation and normalization 6

Data pre-processing and quality checking 8

NPX data quality control 9

Global proteomic analysis across sex, ancestry and technical factors 15

Comparison of Olink proteins with independent assays in UKB 19

Correlation between same proteins measured across panels 20

Proteomic associations with population health components 21

***Proteomic associations with age, sex and BMI* 21**

***Proteomic associations with health burden and prevalent diseases* 22**

***Proteomic associations with renal and liver function markers* 23**

Consequences of high impact pQTLs 24

List of previous pQTL studies 25

Identification, fine mapping, and colocalization of independent signals - further details 27

Biological enrichment for proteins with multiple trans associations 29

Trends of pQTL associations with increasing sample size and proteins assayed 30

Sensitivity analyses of pQTLs 32

Co-localization with expression QTLs 37

ABO blood group and FUT2 secretor epistasis effects provide insights into GI pathophysiology 38

Proteomic insights into pathways underlying COVID-19 associated loci 40

Insights into inflammasome pathways 42

PCSK9 MR with extended instruments expands mirroring of clinical trial pharmacological effects on cholesterol and indicated diseases 44

UKB-PPP consortium banner contributors 46

References 51

Overview of the UKB-PPP study

A schematic overview of the UKB-PPP study is summarised in **Figure S1**. We provide additional details in the sections below.

Figure S1. Schematic overview of the UKB-PPP analysis.

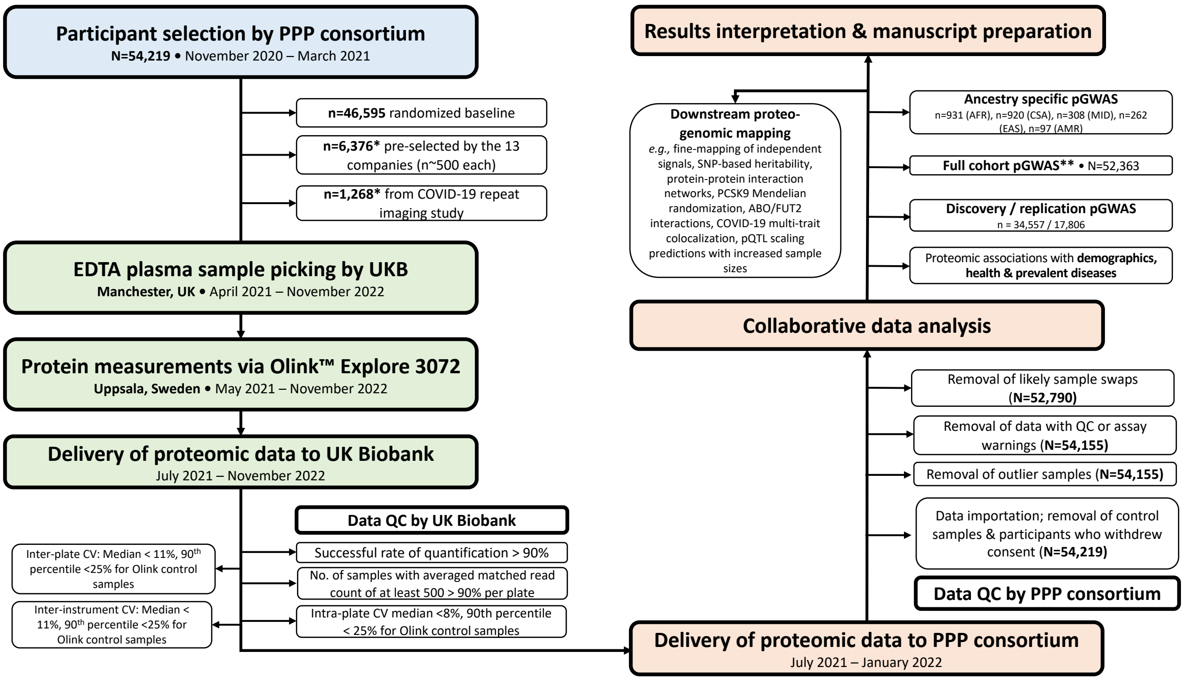

PPP = Pharma Proteomics Project. UKB = UK Biobank. CV = coefficients of variation. *20 participants were both pre-selected by the consortium members *and* participated in the COVID-19 repeat imaging study. **Sample size of the full cohort GWAS reduced from n=52,790 to n=52,363 following removal of samples not measured at baseline visit, without covariate (Methods) or without genetic array/principal component information.

UK Biobank sample selection for UKB-PPP

Samples were selected in two temporally and algorithmically separated picking processes. For the first, initially 5,500 samples collected from participants during their baseline recruitment visit were pre-selected by the Consortium members. 44,502 further representative participant samples were selected from the UK Biobank (UKB) cohort through a stratified selection against age, sex, and recruitment centre for baseline samples, optimised to reduce number of plates for the picking process. For the randomised sample selection, sample picking is stratified using an algorithm which accounts for current storage location of each rack, described in detail in [1], which is also employed in other projects in UKB. In brief, plasma samples from UKB's ~500,000 participants are spread over 21,000 racks each containing samples from 24 participants on average. A rack retrieval from storage takes 12x longer than a tube pick, therefore the most efficient method for picking is to maximise number of samples picked from

each rack in storage. 6,000 samples were preselected by consortium members; the remaining samples were selected from racks visited by consortium picking, with stratified sampling used to ensure a representative distribution through the UK Biobank cohort. Additionally, UK Biobank apply a pseudo-randomisation algorithm to maximise variation of phenotypes on each generated sample plate. Typically, 4 racks (24 samples per rack) are used to generate a rack of 96 picked samples. The algorithm works to ensure the racks are selected in an order that avoids clustering of racks from the same timepoint/sample collection clinic. This in turn results in pseudo-randomisation of other phenotypes like age and gender. Day of week of collection, self-reported participant ethnicity* and deprivation index for selected participants were confirmed as representative of cohort distributions. This created a total selection of 50,002 samples. For the second picking process a total of 7,000 samples were supplied. First, 1,020 pre-selected samples from the Consortium members were chosen. A further 3,637 samples were selected from participants attending the COVID-19 case-control imaging study. 1,270 participants formed this section of the study, who had been invited to attend UK Biobank for a repeat imaging assessment on the basis of a COVID-19 diagnosis from linked healthcare records or a positive home-based SARS-CoV-2 antibody lateral flow test provided by UKB, or were invited as matched controls of these COVID-19 cases. Full inclusion criteria are described in https://biobank.ndph.ox.ac.uk/showcase/showcase/docs/casecontrol_covidimaging.pdf.

For these 1,270 participants, samples from initial recruitment, pre-COVID and post-COVID imaging visits were included where possible. An additional 2,343 baseline samples were included in this picking process selected randomly as described for the first sample selection, optimised to include selection locations required for the COVID imaging and consortium-selected samples.

*We note that self-reported participant ethnicity was collected at each UKB Assessment Centre via touchscreen questionnaires, asking participants, "What is your ethnic group?" and "What is your ethnic background?" These questions were dropped from the touchscreen protocol on October 24th, 2016, as noted in UKB Data Field 21000. We have retained the terms "ethnicity" and "ethnic background" when referring to the touchscreen questionnaire responses; however, all primary analyses described in this manuscript refer to genetic ancestry.

UKB sample handling

EDTA (9ml) vacutainers were collected and fractioned to 850µl aliquots of EDTA plasma, buffy coat and red cells as per the UK Biobank collection protocol described in[2]. Aliquots were stored in an automated -80°C sample archive. EDTA plasma from participants selected for the study were withdrawn from the automated sample archive in a quasi-randomised order[1]. Prior to processing, aliquots were stored in a -80°C freezer. Racks containing 85 aliquots were thawed (column 12 plus one additional random well left empty for controls and two spaces for blinded duplicates, **Figure S2**) and 60µl of EDTA plasma was pipetted to a PCR plate (P/N AB0800, Thermo Scientific™) using a TECAN freedomEVO with full sample tracking. Two blinded duplicates (using aliquots from the same plate) were added to each plate before plates were sealed with adhesive seals (P/N 4306311, Applied Biosystems™) and stored at -80°C. Samples were shipped on dry ice to Olink Analysis Service in Sweden for analysis accompanied by an electronic sample manifest containing sample level information.

Plasma profiling using the Olink technology

The Olink technology uses Proximity Extension Assay, where a matched pair of antibodies labelled with unique complimentary oligonucleotides (proximity probes) bind to the respective target protein in a sample. As a result, the probes come into close proximity and hybridize to each one, enabling DNA amplification of the protein signal, which is quantified on a next generation sequencing read-out which has been described in detail previously[3,4]. Antibodies targeting 2,923 unique proteins are distributed across eight 384-plex panels and each panel is composed of four dilution blocks to accommodate for the different dynamic ranges of target proteins in plasma and serum. The panels focus on inflammation (INF and INF II), oncology (ONC and ONC II), cardiometabolic (CAR and CAR II) and neurological (NEU and NEU II) proteins. A summary of all proteins is shown in **Supplementary Table 3** and a schematic of the assay approach is summarised in **Extended Data Figure 1**. The performance of each protein assays is validated based on specificity, sensitivity, dynamic range, precision, scalability detectability and endogenous interference[3].

Samples were serially diluted to 1:1, 1:10, 1:100, 1:1000, 1:10000, transferred to 384-well plates consisting of four abundance blocks for each of the eight panels per 96 samples. In the immune-reaction, plasma samples were incubated overnight at 4°C with the proximity probes. Oligonucleotides in close proximity are extended and amplified using DNA polymerase and

create a DNA sequence which is amplified by polymerase chain reaction (PCR 1), to create amplicons encoding protein assay information. All amplicons for each sample from the four abundance groups per panel are combined, resulting in one well of amplicons for each sample/panel. Four unique 96- index plates are added to every 4 sample plates, followed by a second PCR (PCR 2), to enable all samples in a plate to be combined in to one library per panel. Each library is bead purified and libraries quality controlled using a Bioanalyzer. Four sample plates from four identical panels were pooled, denatured and sequenced on individual lanes on the Novaseq600 using S4 flow cells v1.5 (35 cycles) and 384-samples and 384-assays measured. Counts of known sequences were translated in to Normalized Protein eXpression (NPX) values within Olink's MyData Cloud Software.

Olink inbuilt assay quality control

The Olink workflow has an inbuilt quality control system, 3 engineered internal controls that are spiked into every sample, and each abundance block. The incubation control (Inc Ctrl) is a non-human assay, green fluorescent protein (GFP) and is used during data QC. The extension control consists of two paired oligonucleotides coupled to an antibody molecule with the DNA arms in close proximity and was used for normalization of the data. The amplification control (Amp Ctrl) consists of a synthetic double stranded DNA template, used for QC and to monitor the PCR steps in the protocol. In addition, each sample plate includes external controls in column 12. A negative control ran in triplicate is used to calculate the limit of detection (LOD) of each assay in every plate, and a plate control sample, consisting of a pooled plasma sample is run in triplicate to adjust the levels between plates. A duplicate pooled sample control is included to estimate precision within and between runs. In each of the four panels Cardiometabolic, Inflammation, Neurology and Oncology, 3 overlapping assays of IL6, IL8 (CXCL8), and TNF are included; in each of the four panels Cardiometabolic II, Inflammation II, Neurology II and Oncology II, 3 overlapping assays of LMOD1, SCRIB and IDO1 are included. Overlapping assays are used for quality control (QC) purposes to assess correlation. Olink's internal QC assessment is performed at two levels; run QC and sample QC. For run QC, each abundance block per panel and sample plate should fulfil the mean absolute deviation (MAD) in both internal controls (Inc Ctrl and Amp Ctrl) which should not exceed 0.3 NPX, the deviation of sample QC level is allowed for up to 1/6 samples and in each panel the median of 90% assays in plate and negative controls should be in the accepted range from predefined values set during validation. The sample QC assesses all samples individually using the internal controls (Inc Ctrl and Amp Ctrl) which should be within ± 0.3 NPX from the plate median

across the abundance block, in addition to the mean assay count for a sample may not be less 500 counts. Samples that do not fulfil these criteria will receive a sample warning for a given abundance block in the data set. Data from these assays should be treated with caution. Assays where the median of the negative Control triplicates deviate more than 5 SDs from predefined values set for each assay during validation will receive a QC warning in the results NPX file. Data was generated according to Olink's standard procedures. Normalized Protein eXpression (NPX) is Olink's relative quantification unit on a log-2 scale. Data generation consists of normalization of matched counts of an assay to the extension control spiked into every sample, log-2 transformation of the data, and level adjustment using the plate control.

NPX calculation and normalization

Samples from the study were divided into two sets: i) Set 1 – UKB; and ii) Set 2 – COVID; depending on the time point they were randomly selected from the UKB population. Samples were randomly assigned to 96-well plates, and fully randomized within plates. Each plate contained: i) 87 samples from set 1 or set 2; ii) 1 empty well; iii) 2 Olink control samples used for quality control; iv) 3 Olink negative control samples used to compute the baseline assay level of each plate; and v) 3 Olink plate control samples used for normalization of protein expression (**Figure S2**). All Olink samples were in column 12 of each plate while the remaining 87 samples + 1 empty well were randomized across columns 1-11 and rows A to H.

Figure S2: Olink 96-well plate layout.

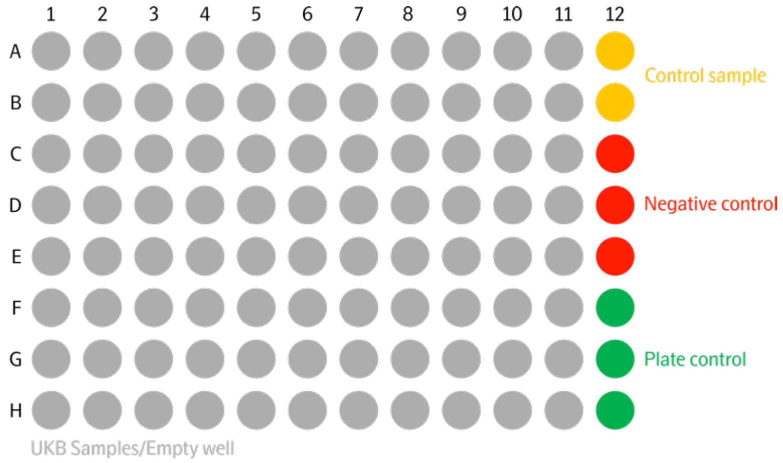

Plates were shipped into 8 batches (0-7) that consisted of different numbers of samples (**Figure S3**). Batches 0-6 contained exclusively samples from set 1, while batch 7 contained samples from both sets. A subset of 25 plates from batch 7 contained exclusively samples from set 1, while 94 of them contained samples from both sets.

Figure S3: Schematic representation of the sample batch design for UKB.

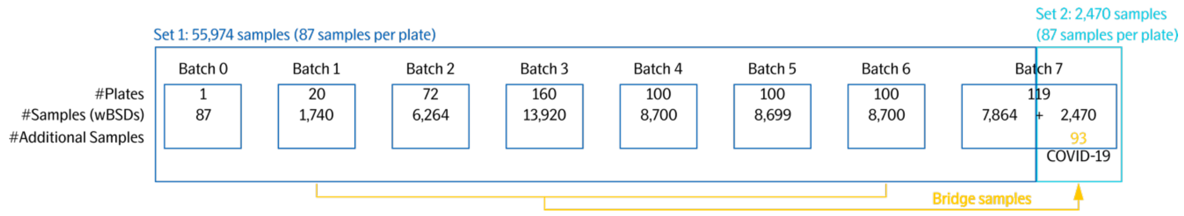

Calculation of Normalized Protein eXpression (NPX) values was performed stepwise as described below. Initially, we calculated the $\log_2$ ratio of counts of each assay of each sample to the counts of the extension control, and the assay-specific median value of the plate controls was subtracted. This provided us with plate normalized NPX values for both sets. For samples in batches 0-6, we subtracted the assay-specific plate median NPX value. 3 assays (PNLIPRP2, TDGF1 and FOLR3) with proven bimodal distribution driven by genotypes[4,5] were excluded from the last step, hence, remained plate-normalized. At this stage data was normalized within each batch. Next, we computed adjustment factors from the difference of the assay specific median NPX value of each batch to the reference batch (batch 1). The selection of the reference batch does not impact coefficients of variation as the across-batches normalization uses differences of assay-specific median NPX values between batches. These are fixed factors in NPX scale that aim at harmonizing median NPX values of assays between batches. Finally, adjustment factors from above were added to the NPX values of each batch of set 1. In summary, set 1 was normalized using a two-step approach of within-batch and across-batches intensity normalization. Samples of set 2 were normalized using reference (bridge) samples that were shared between the two sets. All plates that contained at least one sample of set 2 were assigned a randomly selected sample from the set 1. 93 samples with missing frequency <10% and representative of the NPX dynamic range were selected from batches 1-6 in set 1. These samples were assigned to empty wells of the 93 plates from batch 7 of set 2. In this case, adjustment factors were computed from the assay-specific median of the pair-wise differences between set 1 and set 2. Adjustment factors were added to the NPX values of set 2. Intensity normalized NPX values for set 1 and bridge normalized NPX values for set 2 consisted of the final set of NPX values that was used for downstream analysis.

Within batch normalization centers data at NPX=0 by subtracting the plate-specific median per assay from all samples and assays in the same plate. Across batches normalization computes a set of adjustment factors as the difference of the assay-specific median NPX values of each batch. The first step accounts for plate-to-plate variation within batch and the second step accounts for batch-to-batch variations within the study. Both steps are shifts of an assay-

specific fixed factor in NPX scale; namely, plate median in the first step and difference of assay-specific medians between batches in the second step. Normalization steps do not affect intra-plate CVs as the exact same factors are applied in both steps. Inter-plate CVs after the first step of normalization improve within-batch CV, while inter-plate CVs after the two-step normalization improve CV for the full dataset.

Data pre-processing and quality checking

The raw UKB-Olink data contained 58,776 samples and 54,221 individuals; taking out control samples, unprocessed samples and participants who have withdrawn from the study reduced the data to 58,353 samples and 54,219 individuals.

Outliers were identified by two approaches applied to each panel of proteins: (1) principal component analysis (PCA), and (2) examining the median and IQR of NPX across proteins by sample. The data used for PCA were the NPX data excluding: (1) control samples, (2) those who withdrew from the study/data were not processed, (3) data points missing NPX values, (4) those missing in covariates such as sex and sampling center. We removed data points with (1) a standardized PC1 (the component that captures the most variation) or PC2 (second largest component) value more than 5 standard deviations from the mean (which is zero in standardized PCA), or (2) a median NPX greater than 5 standard deviations from the mean median, or an IQR of NPX greater than 5 standard deviations from the mean IQR. We excluded outliers, data points with a QC or assay warning, as well as likely sample swaps where we excluded the sample across all panels if half or more panels were affected; the remaining data contained 56,695 samples and 52,790 individuals. Suspected sample swaps were identified using inconsistency from proteomic predicted sex and outliers from *cis* pQTLs where we summed the standardized squared residuals over all proteins for each individual and divided by the sum of squared protein levels - samples with the wrong genotype should have larger values than those with correct genotypes. We excluded 1 protein with high degree of QC failures (GLIPR1) from our main analyses. We did not remove data based on LOD (lower limit of detection). We did not perform further processing of the resulting NPX data. The attrition of data is summarized in **Supplementary Table 31**:

Supplementary Table 31. Summary of data attrition during QC.

Number of data points*	% of data with QC/assay warnings	% outlier	% likely sample swap
**171,377,949**	**3.3%**	**0.7%**	**2.6%**

*After removing Olink control samples and those withdrawn from the study.

NPX data quality control

Protein coefficients of variation (CVs)

Two sets of duplicate samples were provided both by Olink Proteomics ('Olink controls') and UK Biobank ('Blind Spike Duplications' / BSDs). We measured the intra-person variability of protein i of individual j on plate k for duplicate samples by calculating the coefficient of variation (CV) of NPX, as recommended by Olink:

$$CV_{ijk} = 100 * sqrt(\exp((log2 * SD(NPX))^2) - 1)$$

Then, to summarize the CV for each protein, we took the median of the CVs across individuals and plates by protein, plotting the median CVs for each protein:

$$median\_CV_i = median(CV_{ijk}) \text{ for each } j \text{ and } k$$

Intra-person CVs of each Olink protein are illustrated in **Figure S4** and shows that the CVs of the proteins range between 1.8% to 27.2%.

Figure S4. Histograms of CVs (coefficients of variation) of proteins on the Olink platform

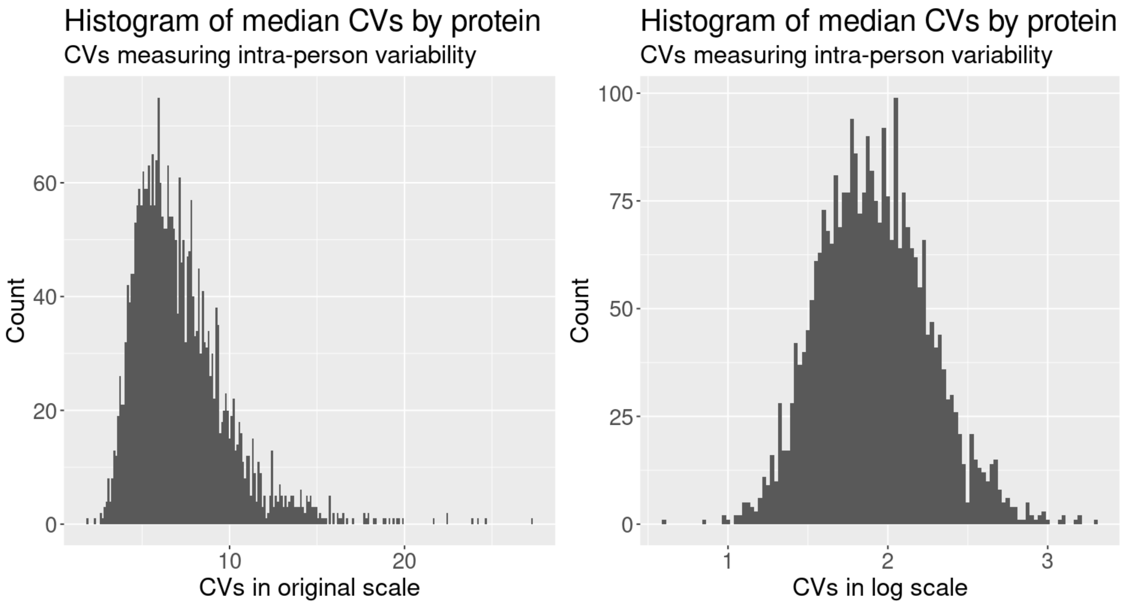

Detecting batch effects

To detect potential batch effects, we fit a simple random-effects model on each protein and calculated the percentage of variability attributable to batch. A high percentage suggested greater variability in NPX across batch. The model used was:

$$NPX = b_0 + u_j + e_{jk} \text{ , where}$$

- b_0 = global mean NPX
- u_j = batch random effects
- e_{jk} = error terms
- Percentage of variability attributable to batch = $\frac{VAR(u_j)}{VAR(u_j+e_{jk})}$

The percentage of variability attributable to batch, by protein, is illustrated in **Figure S5.** Overall, the proportion of variability attributable to batch was low. No notable evidence of batch effects was observed.

Figure S5. Percentage of variability attributable to batch by protein

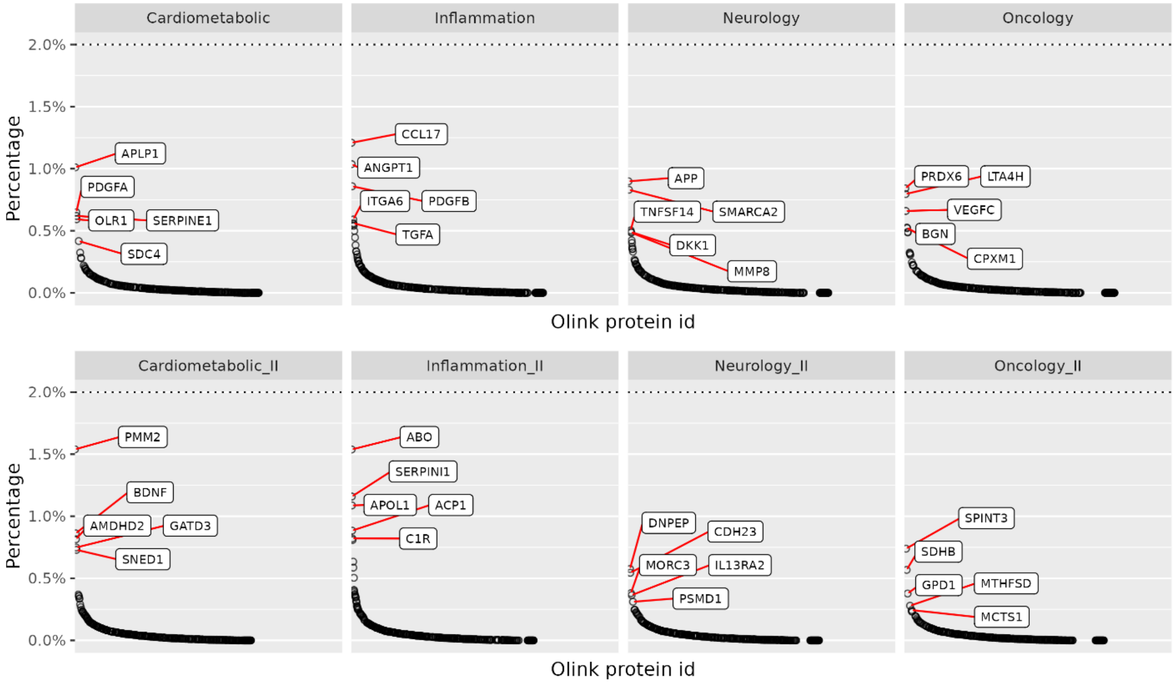

Detecting plate effects

We calculated two sets of CVs for each plate, plotting one series of CVs against the other, and identified whether any plate had high CVs on either axis of the plots. The two sets of CVs were defined as follows:

Approach 1 - CVs measuring inter-replicate variability:

1. Restrict the analysis to the Olink control samples, and calculate the CV by OlinkID and plate
2. Restrict the analysis to the BSD samples (same patient with two samples on the same plate), and calculate the CV by OlinkID and plate
3. Combine data from 1 and 2, and calculate the median of 1 and 2 by OlinkID and plate
4. Calculate the median across OlinkIDs by plate

Approach 2 - CVs measuring inter-patient variability, including all patient samples but not Olink control samples):

1. Calculate the CV by OlinkID and plate
2. Take the median CV across OlinkIDs by plate

We plotted the median CVs from approach 2 against approach 1. If any plate exhibited high CVs on either axis, we examined possible contributions towards the higher CV by:

1. Plotting the histograms of plate median CV, confirming that the plate(s) had a median CV that was not in range with others
2. Within the plate(s) with high CV, plotting the distribution of NPX by sample, to check whether the high median CV was driven by specific samples.

Inter-person versus inter-replicate variability for all plates is illustrated in **Figure S6**, and there is no obvious plate whose CV is out of range of others. Thus, we conclude that no evidence of plate effect is observed.

Figure S6. Plotting CV measuring intra-person vs inter-replicate variability for all plates.

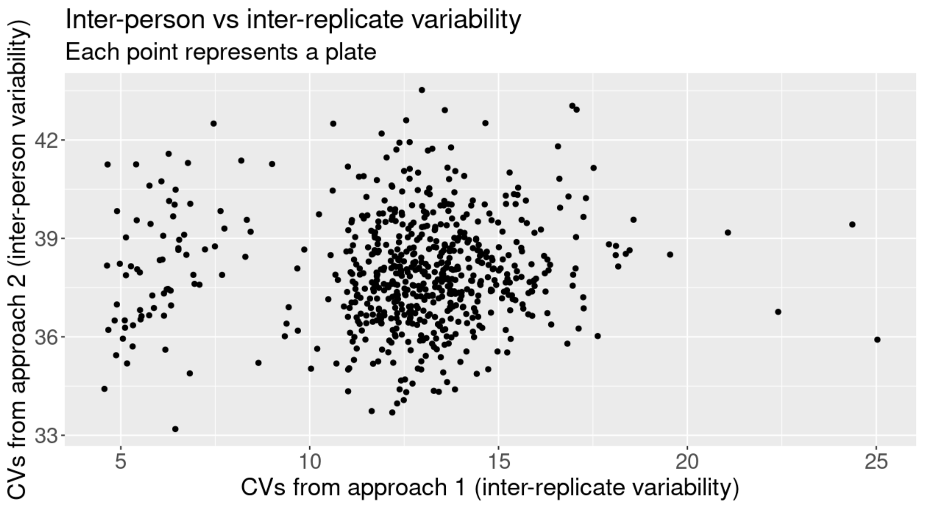

We performed another analysis to examine potential plate effects as we did for batch effects; that is, we fit a simple random-effects model on each protein and calculated the percentage of variability attributable to plate. A high percentage suggested greater variability in NPX across plate, indicating the presence of plate effect. All except those labeled in the plot had inter-class correlation (percentage of variability attributable to plate) below 10%, and we did not think there was a systematic plate effect (**Figure S7**).

Figure S7. Percentage of variability attributable to plate by protein.

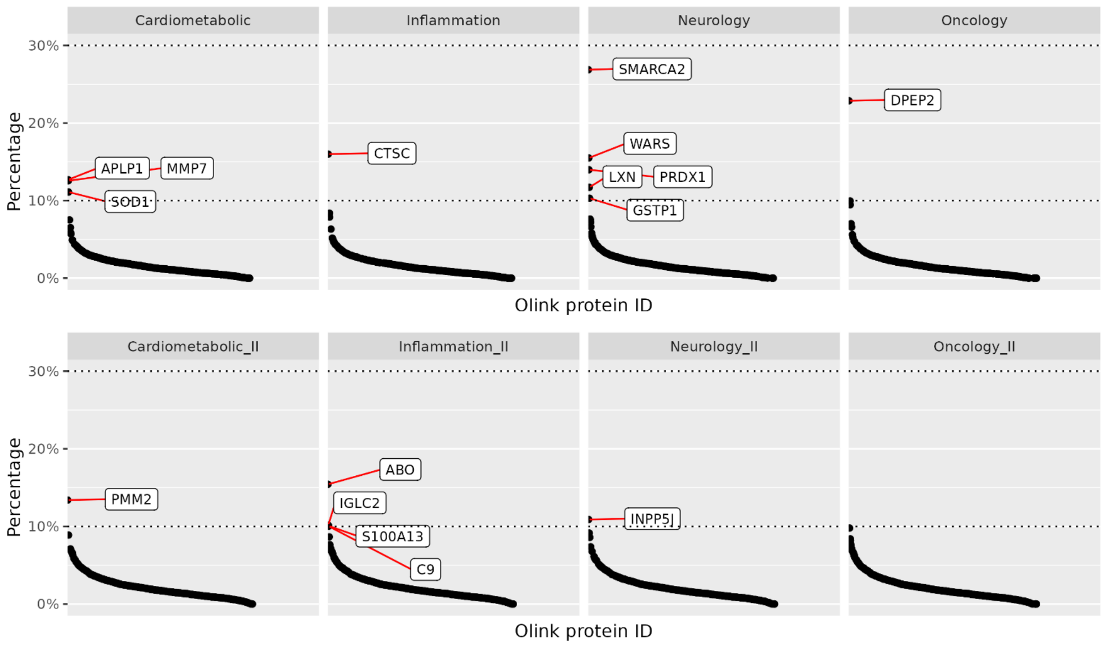

Proteins below limit of detection (LOD)

The proportion of samples with measurements below LOD for proteins across the panel are summarised in **Figure S8.** Most of the protein analytes (2,154 out of 2,941) have the proportion of samples below LOD lower than 30%. 1,981 proteins have the proportion ranged between 0-10%; 92 and 81 proteins ranged between 10-20% and 20-30%, respectively. 787 proteins have 30% or more data falling below the lowest level of detection (**Figure S8a)**.

We also examined how many proteins had less than 5% of samples below LOD, overall and by panel. 1894 proteins, out of 2941, had less than 5% of samples with NPX measurements below LOD. The proportion differed by panel, though. 90.8%, 82.6%, 77.9%, and 81.5% of proteins in Cardiometabolic, Inflammation, Neurology and Oncology panels had <5% samples with NPX measurements below LOD; the corresponding figures for Cardiovascular_II, Inflammation_II, Neurology_II, and Oncology_II panels were 51.2%, 64.4%, 34.2%, and 32.3%, respectively (**Figure S8b**).

Figure S8. (a) Proportions of Samples with NPX measurements below LOD by panel. (b) Proportions of samples with NPX measurements below LOD by panel, after removing proteins with less than 5% below LOD.

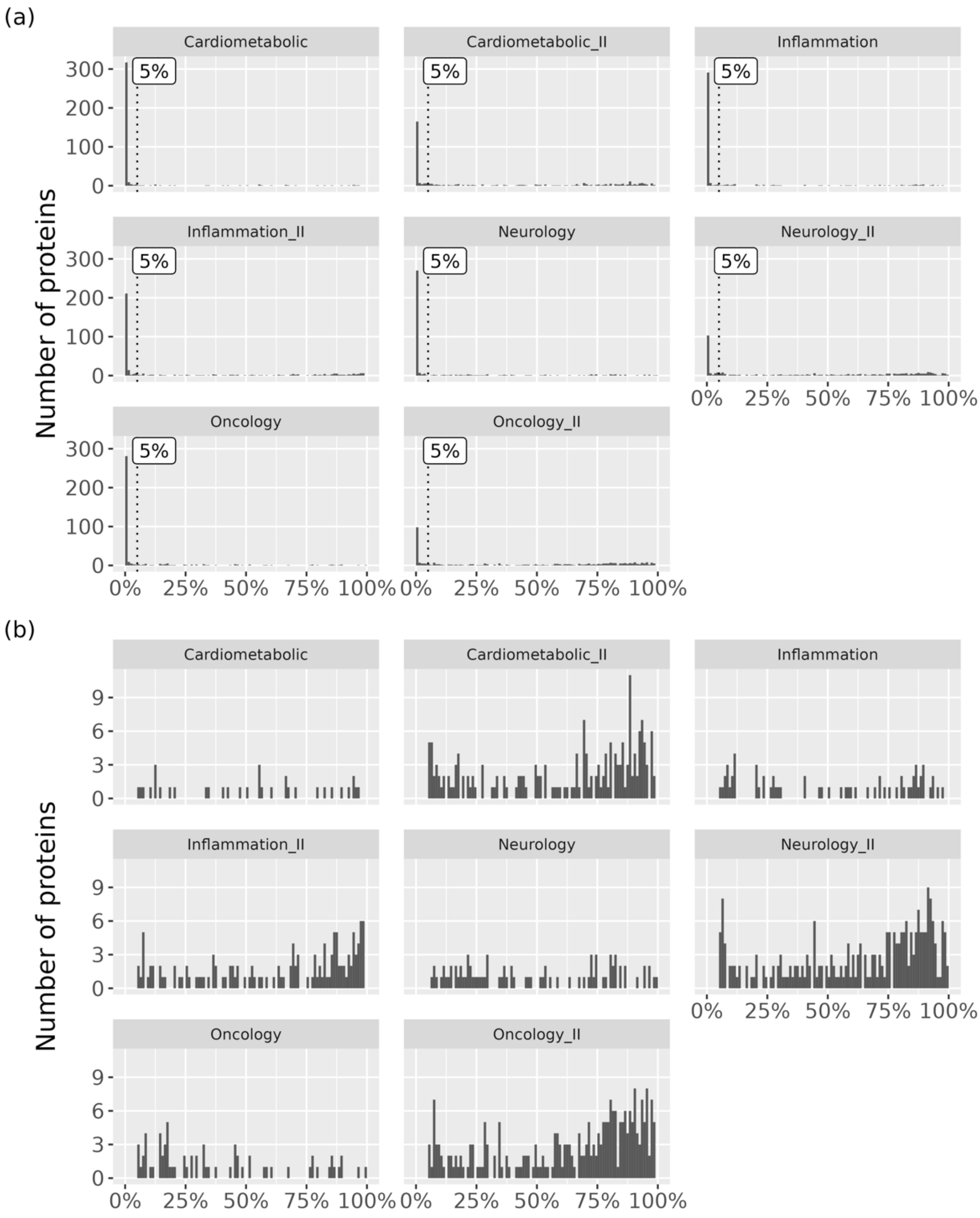

Global proteomic analysis across sex, ancestry and technical factors

In addition to the analyses above, we also performed PCA analysis on the global proteomics data and investigated for any systematic clustering due to sex, ancestry and sampling centre. We also visualize sex and ancestry effects by UMAP to investigate any extreme hidden clusters.

Sex effects

We plotted the global PCA and UMAP results, coloring each data point by sex of the individual; each data point represented a sample. We did not observe clustering of data points of either sex; no global sex effect was indicated.

Figure S9. Proteomic PCA and UMAP colored by sex

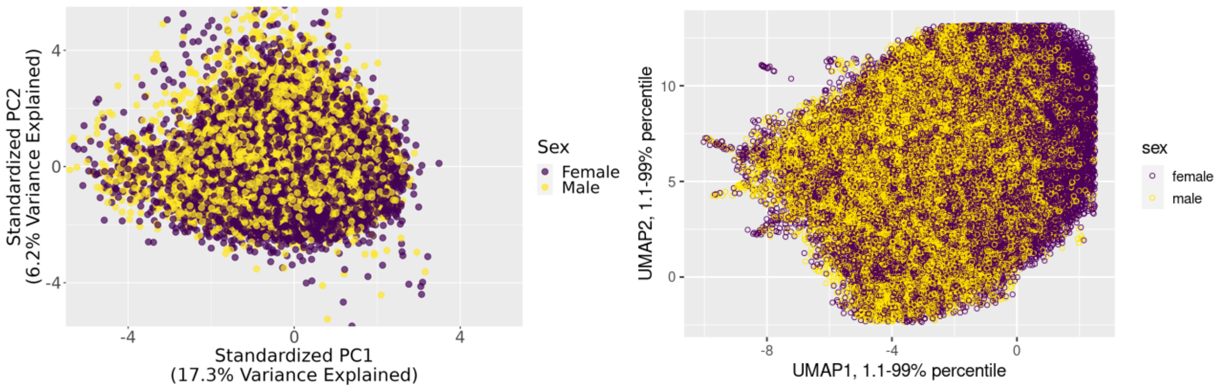

Ancestry effects

The fact that there were six ancestry groups, in which those of European origin accounted for a vast majority of data points, made it difficult to detect any clustering visually. Thus, we separated those of European heritage from others and made two PCA and UMAP plots. We did not observe any clustering of data points in either plot indicating minimal ancestry effect.

Figure S10. PCA and UMAP plot for those of European origins only and other ancestries.

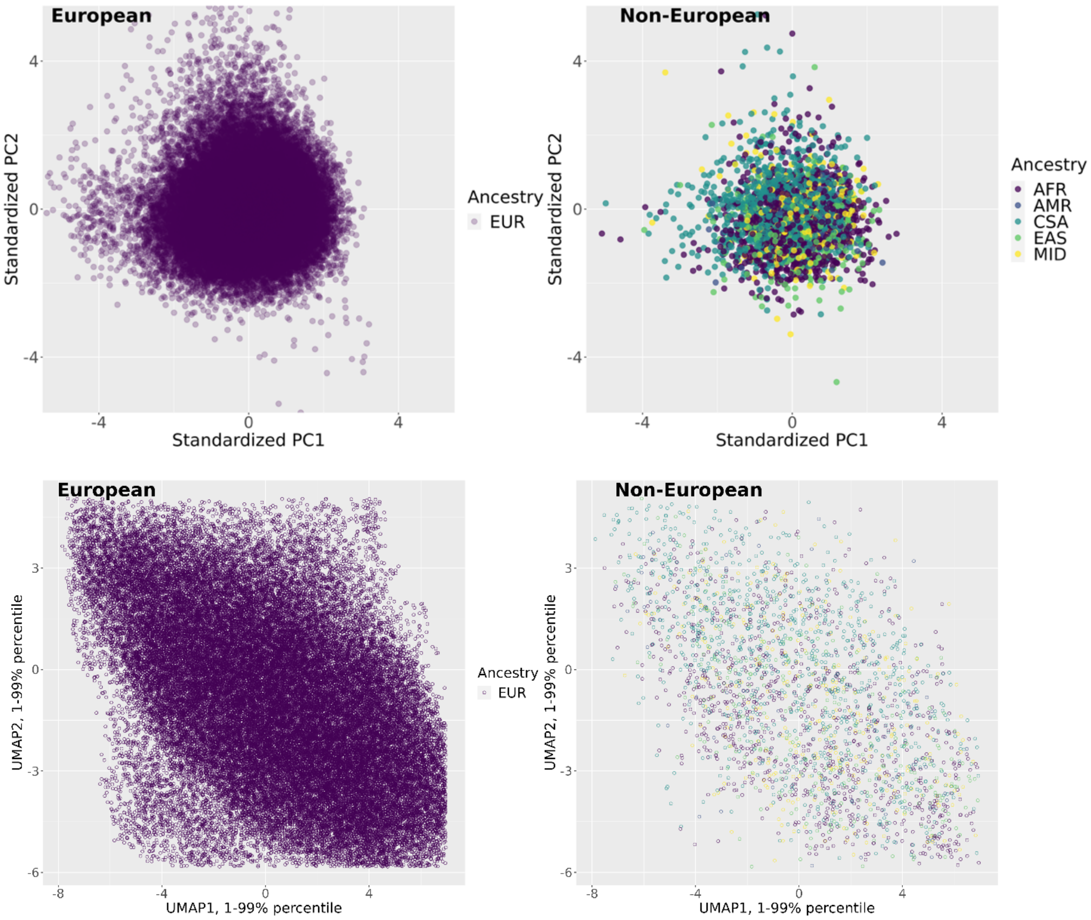

Sampling center effects

To examine potential sampling center effects, plotting the PCA, we examined the distribution of first principal component (PC1) and second principal component (PC2) of each center (**Figure S11**). The median of PC1 and 2 were within 2SD of the means of all centre medians for PC1 and 2. We also took an approach similar to as for batch effect investigation, i.e., fit a simple random-effects model to examine the proportion of variation attributable to sampling center:

$PC_i = b_0 + u_j + e_{jk}$, where

- i = 1 or 2, indicating standardized principal components 1 (PC1) or 2 (PC2)
- b_0 = global mean
- u_j = sampling center random effects
- e_{jk} = error terms
- Percentage of variability attributable to sampling center = $\frac{VAR(u_j)}{VAR(u_j+e_{jk})}$

For PC1, the proportion of variability attributable to sampling center was 3.5%, and the corresponding figures for PC2 was 1.0%. Because the proportion of variability attributable to sampling center was low, we do not think there was strong evidence indicating the existence of sampling center effect.

Figure S11: Distribution of PCs 1 and 2 (the components that explain the largest share of variation) by UKB sampling centers. Sample numbers for each center are at the top. Each box plot presents the median, first and third quartiles, with upper and lower whiskers representing 1.5x inter-quartile range above and below the third and first quartiles respectively.

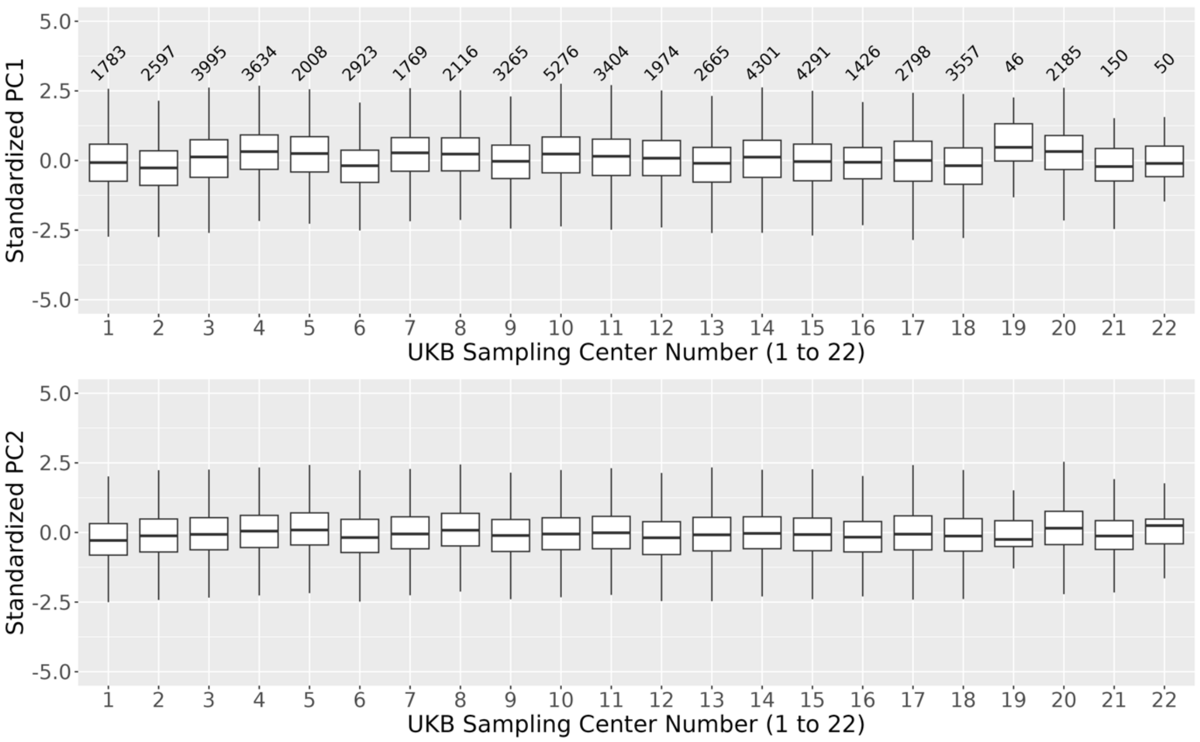

We did not see a particular center that is out of range of others, and hence we did not believe a sampling center effect was indicated.

Sample age effect

Sample age is defined as the difference between the time blood was taken and the protein measurement. Because sample age is a continuous variable (measured in days in its original scale), it may not be very effective to make a PCA plot to detect potential clustering of data as we did for sex or sampling age. Instead, we examine potential "sample age effect" using an approach like batch effect; that is, we fit a simple random-effects model on each protein and calculated the percentage of variability attributable to age of sample Overall the plots show that for most proteins, the percentage of variability in NPX attributable to age of sample is

below < 10% except those labeled on the plots (**Figure S12, Supplementary Table 3**). Thus, we do not think a "sample age effect" is indicated globally.

Figure S12. Percentage of variability attributable to sample age by protein

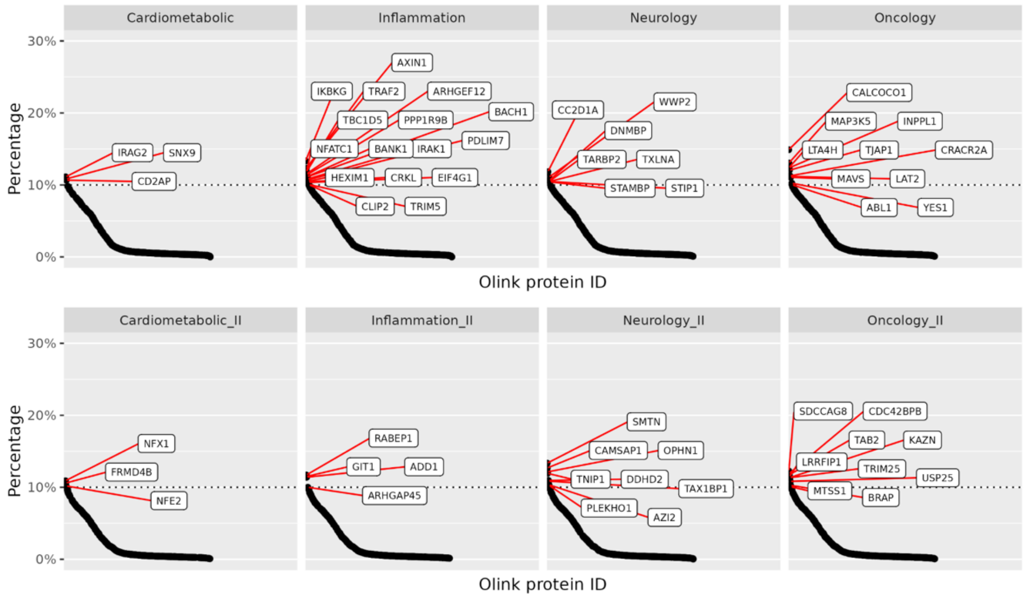

For proteins with >10% variability attributable to sample age (**Supplementary Table 3**), we performed GO enrichment analysis with all proteins tested as background[6], and found significant enrichment (FDR<0.05) for I-kappaB kinase/NF-kappaB signaling (6.9x enrichment ratio, p=2.6x10^{-7}) and regulation of RNA metabolic process (2.4x enrichment ratio, p=9.2x10^{-6}) biological processes; MAPK binding (17.4x enrichment ratio, p=5.1x10^{-4}), ubiquitin-protein transferase activity (9.0x enrichment ratio, p=1.8x10^{-4}), transcription regulatory region sequence-specific DNA binding (5.2x enrichment ratio, p=3.2x10^{-4}), protein domain specific binding (4.0x enrichment ratio, p=2.3x10^{-5}) molecular functions. These are consistent with processes such as MAPK signaling[7] and ubiquitin pathways[8] being extensively implicated in cellular stress.

We found higher sample age contributions to protein variabilities are also correlated to lower proportion of proteins with measures below lower limit of detection (Spearman's ρ=-0.18, p=6.8x10^{-22}), suggesting no evidence of sample-age susceptible proteins being less reliable or abundant than more sample-age stable ones. Storage time effects may vary protein to protein as each protein has its own unique stability, thus care is still needed to account for these effects in corresponding analyses.

Comparison of Olink proteins with independent assays in UKB

We compared seven protein measures acquired using the antibody-based Olink assay with seven corresponding protein measurements obtained using independent assays in UKB, including: Alkaline phosphatase [ALP], apolipoprotein A [APOA1], apolipoprotein B [APOB], aspartate aminotransferase [AST], cystatin C [CST3], lipoprotein(a) [LPA], and sex hormone binding globulin [SHBG]. We found significant positive correlations (max $p<9.8\times10^{-72}$) between Olink and UKB assays (median Spearman's r=0.72, range=0.08-0.94) with higher correlations (median r=0.82, min r=0.67) observed after excluding partial matches due to isoform differences (**Supplementary Table 4**).

Correlation between same proteins measured across panels

Two sets of 3 proteins (CXCL8, IL6, TNF and IDO1, LMOD1, SCRIB) were assayed across four protein panels: CXCL8, IL6, TNF on the first set of Olink protein panels and IDO1, LMOD1, SCRIB on the version II of the respective protein panels (**Supplementary Table 3**). We observed reasonably strong correlations between measurements across panels for each of the 3 proteins measured on each set of four protein panels (**Extended Data Figure 2a**), with mean correlations of r=0.96 for CXCL8 (range: 0.95-0.98), r=0.92 for IL6 (range: 0.88-0.95), r=0.81 for TNF (range: 0.79-0.84), r=0.96 for IDO1 (range: 0.95-0.96), r=0.82 for LMOD1 (range: 0.78-0.90) and r=0.95 for SCRIB (range: 0.94-0.96) – suggesting good reproducibility across protein panels.

Proteomic associations with population health components

Proteomic associations with age, sex and BMI

In total, we found 1,944, 2,092 and 2,348 associations between protein levels and age, sex, and BMI (as covariates in the same model, **Methods**) respectively, at a Bonferroni-corrected threshold of $p<1.7\times10^{-5}$ (**Extended Data Figure 3a, Supplementary Table 5**). Many of the largest observed effects of protein levels with age, sex and BMI were either well-established or repeatedly reported in prior studies[4,9-13] – such as those between age and levels of elastin, GDF15, CHRDL1, and EDA2R; sex and leptin, pregnancy zone protein, prostate specific antigen, and CGA; and BMI and leptin, IGFBP1 and IGFBP2 (**Extended Data Figure 3a, Supplementary Table 5**). We also compared association results between overlapping proteins measured using the aptamer-based SomaScan or Olink assays, where available, for age, sex and BMI in prior published studies[4,9,11,14,15]. Despite differences in population, sample processing and covariate adjustments, we found significant correlations for proteomic effects on age, sex and BMI (max $p=1.8\times10^{-47}$, $r_{age}=0.43-0.55$, $r_{sex}=0.50-0.80$, $r_{BMI}=0.61-0.85$, **Extended Data Figure 3b**). We observed stronger correlations in association effect sizes for overlapping proteins measured using the same Olink assay technology ($r_{Olink}=0.80-0.85$) as opposed to SomaScan ($r_{SomaScan}=0.43-0.73$) (**Extended Data Figure 3b).**

We also explored interaction effects between age, sex and BMI on protein levels in the same model. In total, we found 40 proteins levels with evidence of significant interactions ($p<1.7\times10^{-5}$) between age, sex and BMI; 1,936 between age and sex; 677 between sex and BMI; and 828 between age and BMI (**Supplementary Table 6**). For example, we found the strongest interactions between age and sex for follitropin subunit beta (FSHB, $p=2.6\times10^{-1113}$) and glycodelin (also known as progesterone-associated endometrial protein, PAEP, $p= 6.5\times10^{-1421}$). Follitropin is a key hormone in female reproductive health; the observed increase in follitropin levels at menopausal age in women only (**Figure 1d**) is a well-established hormonal change[16]. Glycodelin is a glycoprotein expressed in mammary glands and endometrial tissues[17]. Levels of glycodelin decreased with age for women only, particularly before the age of menopause (~50 years), whilst for men, levels minimally increased with age (**Figure 1d)**. After 55 years of age, levels of glycodelin minimally increased in women at a similar rate to men. These effects are consistent with the role of glycodelin in female reproductive tissues and their associated changes in hormone levels (such as progesterone) around menopause[17] -

demonstrating that the proteomic assay used in this cohort can capture longitudinal physiological effects from cross-sectional measurements .

Proteomic associations with health burden and prevalent diseases

We investigated the effects of smoking, medication use and the top 20 most prevalent conditions on protein levels (**Supplementary Table 2**), adjusting for age, sex and BMI (**Methods**). We observed widespread patterns of protein dysregulation across 17 of the 20 most prevalent illnesses in UKB, ranging from 35 proteins associated with other dermatitis (ICD-10: L30) to 1,339 proteins associated with essential hypertension (I10), as summarized in **Supplementary Table 7**. Notably, we observed some of the largest associations between disorders of lipoprotein metabolism (E78) and proprotein convertase subtilisin (PCSK9, beta=0.52; p=1.1x10^{-558}), chronic ischemic heart disease (I25) and NT-proBNP (beta=0.51; p=1.73x10^{-310}), diaphragmatic hernia (K44) and chromogranin-A (CHGA, beta=0.48; p=1.51x10^{-276}), gastro-esophageal reflux disease (K21) and pepsin A-4 (PGA4, beta=0.48; p=2.9x10^{-327}), gastritis and duodenitis (K29) and gastrin (GAST, beta=0.36; p=4.15x10^{-175}), and unspecified osteoarthritis (M19) and collagen alpha-1 (COL9A1, beta=0.32; p=5.22x10^{-158}) – all of which demonstrate clear biological links between the protein and disease.

Growth differentiation factor 15 (GDF15), a candidate diagnostic biomarker for mitochondrial disorders frequently induced under stress conditions[18], showed significant associations with 18 of the 20 most prevalent illnesses in UKB and number of medications (p= 1.7x10^{-869}), independent of age. A total of 1,212 proteins were associated with smoking status; CXCL17, a major chemotactic factor for lung macrophages[19], was the most significant (beta=0.49; p=1.7x10^{-865}). Smoking-associated proteins were enriched for expression in the lung (fold enrichment [FE]=1.64, p=4.5x10^{-5}), and included several candidate cancer biomarkers, such as carcinoembryonic antigen-related cell adhesion molecule 5 (CEACAM5, beta=0.34; p=3.98x10^{-317}) and mesothelin (MSLN, beta=0.3; p=1.12x10^{-271}). Other notable findings, including widespread upregulations of inflammatory markers in participants with depressive episodes (F32) and increased concentrations of cartilage-, muscle- and immune-related proteins in joint disorders (M25), are detailed in **Supplementary Table 7**.

We note that all proteomic associations with health burden and disease have not been adjusted for the potentially complex influences of disease severity or time between diagnosis and blood

collection; thus, these observational findings should be interpreted carefully, and downstream validation studies are strongly recommended.

Proteomic associations with renal and liver function markers

We investigated proteomic associations with estimated glomerular filtration rate (eGFR) – a measure of renal function, and liver function enzymes alanine transaminase (ALT) and aspartate transaminase (AST) (**Methods**). We identified 1,815 proteins significantly associated with eGFR (p<1.7x10^{-5}). As anticipated, CST3 was most strongly associated with eGFR, (**Supplementary Table 7)**, owing to its use in the calculation of eGFR values[20] via the CST3 immuno-turbidimetric assay (**Supplementary Table 4)**. We also found 16 assayed proteins that have previously been associated with end stage renal disease[21] to be significantly associated with reduced eGFR (max p=1.8x10^{-17}). For liver function, 2,016 and 1,843 proteins were associated with ALT and AST, respectively, and 18 of the top 20 associations with ALT/AST were enzymes (**Supplementary Table 7**). The most strongly associated non-enzymatic protein for both ALT and AST was KRT18, which is abundantly expressed in the liver and released in liver cell death, and serves a biomarker for liver disease[22]. We found that proteins associated with ALT (fold enrichment [FE]=1.23, p=1.9x10^{-5}) and AST (FE=1.33, p=7.6x10^{-8}) were enriched for expression in the liver; this enrichment became more pronounced when limited to the top 20 associations with ALT or AST, respectively (FE=13.2, p=4.5x10^{-12}).

We trained LASSO models with 10-fold cross-validation to determine whether plasma proteomics can be used to infer demographics (age, sex, BMI), renal (eGFR) and liver (ALT, AST) functions as well as blood groups (**Methods**). Evaluating the models on 20% random held-out data, we found that proteomic data alone provided good predictive performance (Pearson correlation r: 0.87-0.90) for all five quantitative traits, as well as for qualitative traits: sex (F1 score=0.998) and for O (F1=0.94), A (F1=0.92) and B (0.92) blood groups (**Extended Data Figure 3c, Supplementary Table 8**). Taken together, these results suggest that plasma proteomic measures can capture biologically relevant relationships and provide meaningful imputations of demographic features and organ functions.

Consequences of high impact pQTLs

Following annotation of the primary pQTLs, we identified 37 *cis* pQTLs annotated as potential high-impact variants, with 33 (89%) associating with lower *cis* protein levels, and all 15 of the primary *cis* pQTLs coding for start codon lost/stop codon gained, associating with lower corresponding protein levels (**Supplementary Table 12**). 24 *trans* pQTLs SNPs were also annotated as potential high-impact (**Supplementary Table 12)**. The majority of pQTLs identified in this study were located at non-coding regions. These non-coding pQTLs were enriched in regulatory regions, including SNPs located at promoters, enhancers, transcription factor binding sites, CTCF binding sites, and open chromatin regions (hypergeometric test $p=3.1 \times 10^{-6}$**; Supplementary Table 13**). Of the *cis* pQTLs, 26% (512) were protein-altering variants, or in LD ($r^2>0.8$) with protein-altering variants (**Supplementary Table 14**). These observations are consistent with findings in recent large scale studies leveraging Olink or Somascan panels[14,23,24].

List of previous pQTL studies

To evaluate whether the pQTLs in the discovery set were novel, we used a list of published GWAS with proteomics (http://www.metabolomix.com/a-table-of-all-published-gwas-with-proteomics/) and the GWAS catalog (https://www.ebi.ac.uk/gwas/) to identify previously published pQTL studies.Metabolomix,com contains an extensive collection of studies that combine genetics with proteomics or multi-omic data – some of which may not be included in the GWAS Catalog. From these two sources, we included studies published before March 15th, 2023. Studies were required to meet the following criteria:

1. Study was a peer-reviewed publication; pre-prints and abstracts were excluded.
2. Study was conducted genome-wide, sourced from genome-wide array, exome sequencing, and/or whole genome sequencing data.
3. Results reported in publication passed genome-wide significance threshold (p<5e-8); studies were excluded if they tested or reported SNPs in candidate genes or only tested/reported SNPs in *cis* genes.
4. Appropriate p-value adjustment (genome-wide threshold) was included and reported; no false discovery rate corrections or studies that fail to report p-value adjustment methods.
5. Phenotypes tested must be protein based: Studies from flow cytometry or other cell imaging modalities were excluded.
6. Studies must report all proteins in a panel. Studies that generated proteomic data on a platform but only reported selected proteins from that panel were not included.

Following this curation, thirty-four studies were included (**Supplementary Table 32)**. Using a *p*-value threshold of $1.7x10^{-11}$, we identified the sentinel variants and associated protein(s) from the previously published studies and queried those against our discovery pQTLs. If a previously associated sentinel variant-protein pair fell within a 1 Mb window of the discovery set pQTL sentinel variant for the same protein and had an r^2>0.8 with any significant SNPs in the region, it was considered a replication.

Supplementary Table 32. List of previously published pQTL studies evaluated

Author	Publication date	PMID	URL	Proteomics technology
Melzer D	2008-05-09	18464913	https://pubmed.ncbi.nlm.nih.gov/18464913/	immunoassay
Johansson A	2013-03-19	23487758	https://pubmed.ncbi.nlm.nih.gov/23487758/	mass spectrometry
Kim S	2013-07-23	23894628	https://pubmed.ncbi.nlm.nih.gov/23894628/	immunoassay
Enroth S	2014-08-22	25147954	https://pubmed.ncbi.nlm.nih.gov/25147954/	PEA/OLINK
Kauwe JS	2014-10-23	25340798	https://pubmed.ncbi.nlm.nih.gov/25340798/	immunoassay
Sun W	2016-08-17	27532455	https://pubmed.ncbi.nlm.nih.gov/27532455/	immunoassay
Ahola-Olli AV	2016-12-13	27989323	https://pubmed.ncbi.nlm.nih.gov/27989323/	immunoassay
Sasayama D	2016-11-26	28031287	https://pubmed.ncbi.nlm.nih.gov/28031287/	Aptamer/Somascan
Suhre K	2017-02-27	28240269	https://pubmed.ncbi.nlm.nih.gov/28240269/	Aptamer/Somascan
Deming Y	2017-02-28	28247064	https://pubmed.ncbi.nlm.nih.gov/28247064/	immunoassay
Folkersen L	2017-04-03	28369058	https://pubmed.ncbi.nlm.nih.gov/28369058/	PEA/OLINK
Carayol J	2017-12-12	29234017	https://pubmed.ncbi.nlm.nih.gov/29234017/	Aptamer/Somascan
Sun BB	2018-06-06	29875488	https://pubmed.ncbi.nlm.nih.gov/29875488/	Aptamer/Somascan
Emilsson V	2018-08-02	30072576	https://pubmed.ncbi.nlm.nih.gov/30072576/	Aptamer/Somascan
Yao C	2018-08-15	30111768	https://pubmed.ncbi.nlm.nih.gov/30111768/	immunoassay
Hillary RF	2019-07-18	31320639	https://pubmed.ncbi.nlm.nih.gov/31320639/	PEA/OLINK
Ruffieux H	2020-06-03	32492067	https://pubmed.ncbi.nlm.nih.gov/32492067/	Aptamer/Somascan
Zhong W	2020-06-23	32576278	https://pubmed.ncbi.nlm.nih.gov/32576278/	PEA/OLINK
Bretherick A	2020-07-06	32628676	https://pubmed.ncbi.nlm.nih.gov/32628676/	PEA/OLINK
Hillary RF	2020-07-08	32641083	https://pubmed.ncbi.nlm.nih.gov/32641083/	PEA/OLINK
Folkersen L	2020-10-16	33067605	https://pubmed.ncbi.nlm.nih.gov/33067605/	PEA/OLINK
Pietzner M	2021-11-12	34648354	https://pubmed.ncbi.nlm.nih.gov/34648354/	Aptamer/Somascan
Ferkingstad E	2021-02-21	34857953	https://pubmed.ncbi.nlm.nih.gov/34857953/	Aptamer/Somascan
Yang C	2021-07-08	34239129	https://pubmed.ncbi.nlm.nih.gov/34239129/	Aptamer/Somascan
Katz D	2021-11-24	34814699	https://pubmed.ncbi.nlm.nih.gov/34814699/	Aptamer/Somascan
Png G	2021-12-02	34857772	https://pubmed.ncbi.nlm.nih.gov/34857772/	PEA/OLINK
Gudjonsson A	2022-01-25	35078996	https://pubmed.ncbi.nlm.nih.gov/35078996/	Aptamer/Somascan
Caron B	2022-09-03	35264221	https://pubmed.ncbi.nlm.nih.gov/35264221/	immunoassay
Surapaneni A	2022-07-21	35870639	https://pubmed.ncbi.nlm.nih.gov/35870639/	Aptamer/Somascan
Katz D	2022-08-19	35984888	https://pubmed.ncbi.nlm.nih.gov/35984888/	Aptamer/Somascan
Thareja G	2022-09-22	36168886	https://pubmed.ncbi.nlm.nih.gov/36168886/	Aptamer/Somascan
Hansson O	2023-01-11	36504281	https://pubmed.ncbi.nlm.nih.gov/36504281/	PEA/OLINK
Xu F	2023-02-16	36797296	https://pubmed.ncbi.nlm.nih.gov/36797296/	mass spectrometry
Koprulu M	2023-03-23	36823471	https://pubmed.ncbi.nlm.nih.gov/36823471/	PEA/OLINK

Identification, fine mapping, and colocalization of independent signals - further details

We identified 29,420 independent pQTL signals with SuSiE regression of individual-level protein levels on genotype dosages and confirmed statistical independence through multiple linear regression models (**Supplementary Table 16**). This included 10,750 and 18,670 signals that mapped to *cis* and *trans* regions, respectively. 87% (1,717/1,967) of *cis* regions contained more than one signal (mean 5.5 signals per *cis* region) (**Extended Data Figure 6a**). For 12 proteins where the underlying gene mapped outside of the MHC, there were 20 or more *cis* signals, including CLUL1, TPSAB1, and PSG1, which had 31, 27, and 26 distinct signals, respectively. By comparison, only 30% (3,719/12,374) of *trans* regions contained more than one signal (mean 1.5 signals per *trans* region). Regardless of whether the regions were *cis* or *trans*, the primary pQTL signals (defined by highest Bayes Factor) were driven by variants that were more common on average (mean MAF for primary signals: 27.2%, secondary signals: 10.3%).

We also performed fine-mapping with SuSiE to narrow down 95% credible sets of causal variants for each pQTL signal (**Supplementary Table 16**). Credible sets contained an average of 20.5 variants (range: 1-3,189) and were generally better resolved for *cis* signals compared to *trans* signals (mean credible set size *cis*: 9.7; *trans*: 26.7) (**Extended Data Figure 6b**). For 8,700 signals, we were able to determine the likely causal variant (top variant PIP $\geq$ 0.95), which was also favored towards *cis* signals (percentage of *cis* signals: 43%; *trans*: 22%). We provide additional results on (1) boosting and masking of independent genetic signals, (2) credible sets containing protein-altering variants and (3) *cis* pQTLs for a protein having additional *cis* or *trans* effects on other proteins below and in **Supplementary Tables 17** and **18.**

Joint tagging between two or more causal variants by another non-causal variant can boost the significance of the non-causal variant in the marginal association[25-27]. We observed evidence for boosting at 3.3% (470) of tested associations, where the sentinel variant from the marginal analysis was not identified in any of the credible sets from the conditional analysis. Strong primary signals can mask the effect of independent signals in the same region, attenuating their significance in the marginal association[28]. We observed evidence for masking at 2.4% (714) of independent signals that were either not significant in the marginal analysis (p>0.05) or had

opposite directions for conditional effects compared to their marginal effects. Together, these results underscore the importance of modelling all variants within an associated region for accurate signal identification.

There were 7,836 credible sets that contained at least one protein-altering variant (PAV) (**Supplementary Table 17**), including 1,828 for signals that mapped to *cis* regions and where the PAV affected the assayed protein. Of these *cis* credible sets with PAVs, 96% (1,751) contained a single PAV which was also frequently the most probable variant (1,420), and the signals underlying them were also more likely to be the primary pQTL signal (percentage of primary signals containing *cis* PAV: 26%, secondary signals: 15%). Several *trans*-acting PAVs mapped to well-established pleiotropic loci, including examples mapping to proteins that were not assayed, such as GCKR p.Leu446Pro, PNPLA3 p.Ile148Met, and SLC39A8 p.Ala391Thr. which were found in the credible sets for 170, 81, and 72 proteins, respectively.

To explore whether the *cis* pQTL signals for a given protein also had *cis* or *trans* effects on other proteins, we performed pairwise colocalization analyses using the coloc with SuSiE framework. Of 10,750 independent *cis* signals, 11% (1,233) were colocalized with at least one signal from another protein (**Supplementary Table 18**), after excluding 42 signals for CXCL8, IDO1, and LMOD1 which were colocalized with themselves across multiple protein panels. Out of 6,840 unique combinations of colocalized signals, 95% (6,515) reflected colocalization between *cis* and *trans* signals, with the remaining 325 reflecting colocalization between *cis* signals of two different proteins. Most of the proteins with reciprocating colocalized *cis* pQTLs mapped to gene clusters, such as *FCGR2/3* (1q23), *AMY1/2* (1p21), and *LILR* (19q13). However, we also observed instances where colocalized *cis* pQTLs had effects on two nearby genes that otherwise appeared unrelated such as *SPP1/PKD2, FURIN/FES* and *TIMP1/CFP*, which could be explained by non-coding variants in enhancers that regulate more than one gene.

Biological enrichment for proteins with multiple trans associations

For pQTLs associated with multiple independent regions (≥5) across the genome, we performed gene-set enrichment analyses by Ingenuity Pathway Analysis (IPA) to identify enrichment of biological functions. We found enriched pathways for 254 proteins (FDR corrected $p<1.7\text{x}10^{-5}$), including numerous enriched pathways in cellular activation, survival and signaling relevant to immune cells (**Supplementary Table 22**). For example, variants mapped to the nearest genes *TNFSF13B, EGFR, PAK2, HLA-DRB1, CR2, TNFRSF13B, RUNX1, ST6GAL1, PAX5* and *FOXO1* were associated with CR2 (complement receptor 2, expressed on B-lymphocytes) levels; these genes were also enriched in the IL8-signalling pathway that activates lymphocytes ($p=9.25\text{x}10^{-10}$) – corroborating the role of B-cell signaling in regulating CR2 and vice versa. In addition, variants mapped to several nearest genes that were associated with APOA1, APOC1, APOF, FGFBP2, PLA2G7 and MXRA8 levels; these genes were enriched in the plasma lipoprotein particle organization, assembly, remodeling, and clearance functions as well as involved in the aortic atherosclerotic lesion formation ($p<1.2\text{x}10^{-10}$).

Trends of pQTL associations with increasing sample size and proteins assayed

We observed an initial increase in detectable *cis* pQTLs at sample sizes below 5,000 before slowly plateauing as the number of *cis* pQTLs trended towards the number of proteins tested – the upper bound. However, *trans* pQTLs continued to increase with larger sample sizes, without signs of plateauing beyond 50,000 participants. Of the four *trans* pQTLs associated with IL15 levels in the IL15 signaling pathway, associations at the *IL15RA, IL2RB, JAK1, JAK3* loci would not have been detected on average ($p<1.7\text{x}10^{-11}$) at sample sizes below 25,000, 15,000, 25,000 and 20,000, respectively – all four would have been missed at a sample size of 5000 even at nominal $p<5\text{x}10^{-8}$ significance. Moreover, of the 6 *trans* associations for MASP1 in the complement pathway, associations at the *MASP2, MBL2, FCN3, COLEC11, SERPING1* and *VTN* loci would not have been detected on average at sample sizes below 5,000, 1,000, 1,000, 1,000, 10,000, 15,000, respectively. Hence, larger sample sizes would likely lead to increased discovery of *trans* pQTLs networks as opposed to isolated *trans* associations.

Mean variance explained by *cis* associations quickly plateaued beyond samples sizes >5,000 whilst the mean variance explained by *trans* associations continued to slowly increase and drive most of the increase in mean total variance explained at sample sizes >5,000 (**Figure 2f**). We found the mean proportion of variance explained by independent pQTLs increased the most at sample sizes less than 5,000 (**Figure 2f**). Mean variance explained by *cis* associations quickly plateaued beyond samples sizes >5,000 whilst the mean variance explained by *trans* associations continued to slowly increase and drive most of the increase in mean variance explained at sample sizes >5,000 (**Figure 2f**).

Overall, the rate of increase in the number of pQTL associations with increasing proteins measured slowed beyond the most abundant 1,000 assayed proteins (**Methods**, **Figure 2g)**. This is consistent with more lower abundance proteins being increasingly sampled and the reduced pQTL detection at lower dilutions (lower expected abundance) observed in this study. Although the yield of new pQTLs is starting to decrease as newer larger assay platforms begin to measure more low abundant proteins (% of proteins in 1:1 dilution section = 76% in Olink II panels *vs* 57% in Olink I panels, **Supplementary Table 3**), we still anticipate considerable additional associations to be gained, especially at large sample sizes, beyond the proteins

measured here (**Figure 2g**), before the saturation point of all detectable proteins present in plasma.

We also found a shift towards an increasing number of genomic regions harboring associations with multiple proteins with larger sample sizes, indicating greater detectability of pleiotropic loci at increased study sizes (**Extended Data Figure 9a**). Furthermore, we found a slightly sublinear increase in the number of proteins with at least one interacting protein in any *trans* loci as sample size increased **(Extended Data Figure 9b)** – suggesting additional *trans* target interacting loci for other proteins can be found with larger studies. However, the proportion of *trans* loci containing at least one interacting protein with the tested protein decreased at a slowing rate with sample size (**Extended Data Figure 9c**) – suggesting increased detections of *trans* loci are not driven by direct protein-protein interactions.

Sensitivity analyses of pQTLs

We also explored, *a priori,* the impact of blood cell composition, BMI, seasonal and fasting time before blood collection on pQTL effects (**Supplementary Table 24, Figure S13**).

Effects of blood cell counts

Most primary associations from the discovery analysis (84.0% [12,007/14,287], including 99.4% [1,943/1,955] of *cis* and 81.6% [10,064/12,332] of *trans* associations) remained significant ($p<1.7\text{x}10^{-11}$) in the blood-cell sensitivity analysis, which adjusted for blood-cell composition (**Methods**). 104 *trans* associations (0.84% [104/12,394]) fell above nominal significance ($p>0.05$) with the addition of blood-cell covariates. Of these 104 *trans* associations, 96 were with a sentinel variant rs1354034 in *ARHGEF3*. In total, the *ARHGEF3* variant rs1354034 significantly associated with 444 proteins in the discovery analysis, and 95 proteins in the blood-cell sensitivity analysis. The *ARHGEF3* locus is established to be highly pleiotropic and known to associate with platelet counts[29,30]. A previous plasma pQTL study suggested that the observed pleiotropy at *ARHGEF3* may be driven by genetically determined increases in platelet counts and related sequelae that cause proteins to be secreted into plasma during sample handling and preparation[29]. We further tested this hypothesis through a formal mediation analysis of 349 variant – protein associations for platelet counts at the *ARHGEF3* locus using individual participant data (Methods). After correction for multiple testing (Bonferroni correction for 349 variant – protein associations and 9 blood cell phenotypes; $p=1.25\text{x}10^{-5}$), 79.08% of the associations were found to be fully mediated by platelet counts, in line with the observed decrease in pleiotropy at *ARHGEF3* variant rs1354034 after adjusting for blood-cell composition.

We cross-referenced the 2,415 proteins with pQTLs for enrichment in various tissues including blood reported by Uhlen *et.al*[31] (**Supplementary Table 24**). Within the list of 2,415 proteins, 3 proteins (CLEC4C, IFNL1 and MAP1LC3B2) were enriched in blood and 34 other proteins were enriched in more than one tissue including blood (**Supplementary Table 33**). The list of these proteins along with the tissues and the specific distributions are detailed in **Supplementary Table 24**.

Effects of BMI

In the BMI-adjusted analysis, all but 1.8% [264/14,287; 259 *trans* associations, 5 *cis* associations] of the primary associations from the discovery analysis remained significant (p<1.7x10^{-11}). Of these 259 *trans* associations, only one association (leptin levels – rs56094641) fell below a significance threshold of p<0.05 (p=0.15). This association with leptin levels was driven by an intronic variant (rs56094641) in *FTO*, an established obesity associated locus, suggesting that leptin association with this variant is mediated by obesity[32].

Effects of season and amount of time fasted at blood collection

The majority (99.4% [14,207/14,287]) of sentinel pQTLs identified by the discovery analysis remained genome-wide significant (p<1.7×10^{-11}) after adjustment for season and participant-reported fasting time at blood collection. *P*-values for the 80 associations (2 *cis*, 78 *trans* pQTLs) that were no longer genome-wide significant after accounting for multiple testing ranged from 5.0×10^{-11} to 1.7×10^{-11}, suggesting minimal impact of season and/or fasting time on variant associations with protein levels.

Proteomic variance contributions by blood cell composition, BMI, seasonal and fasting time

We additionally explored the effects of the aforementioned factors along with demographics contributing to the proteomic variance explained (**Figure S14, Supplementary Table 25**). We first created a reference model regressing the demographic covariates used in GWAS (age, gender, age*gender, age^2, age2*gender, ancestry and 20 genetic PCs) on protein levels. We then calculated the difference in the variance explained by the reference model and a multivariable model including the base covariates and each of the non-genetic factors separately to evaluate the percentage of variance in protein levels explained by the non-genetic factors alone. On average, demographic covariates explain 3.7% of the variance in plasma protein levels, while blood cell covariates, BMI, fasting time, and season explain 4%, 1.5%, 0.06%, and 0.03% respectively.

Figure S13. Comparison of effect size and p-value changes before and after sensitivity analyses. *P*-values derived from REGENIE regression GWAS (two-sided, unadjusted).

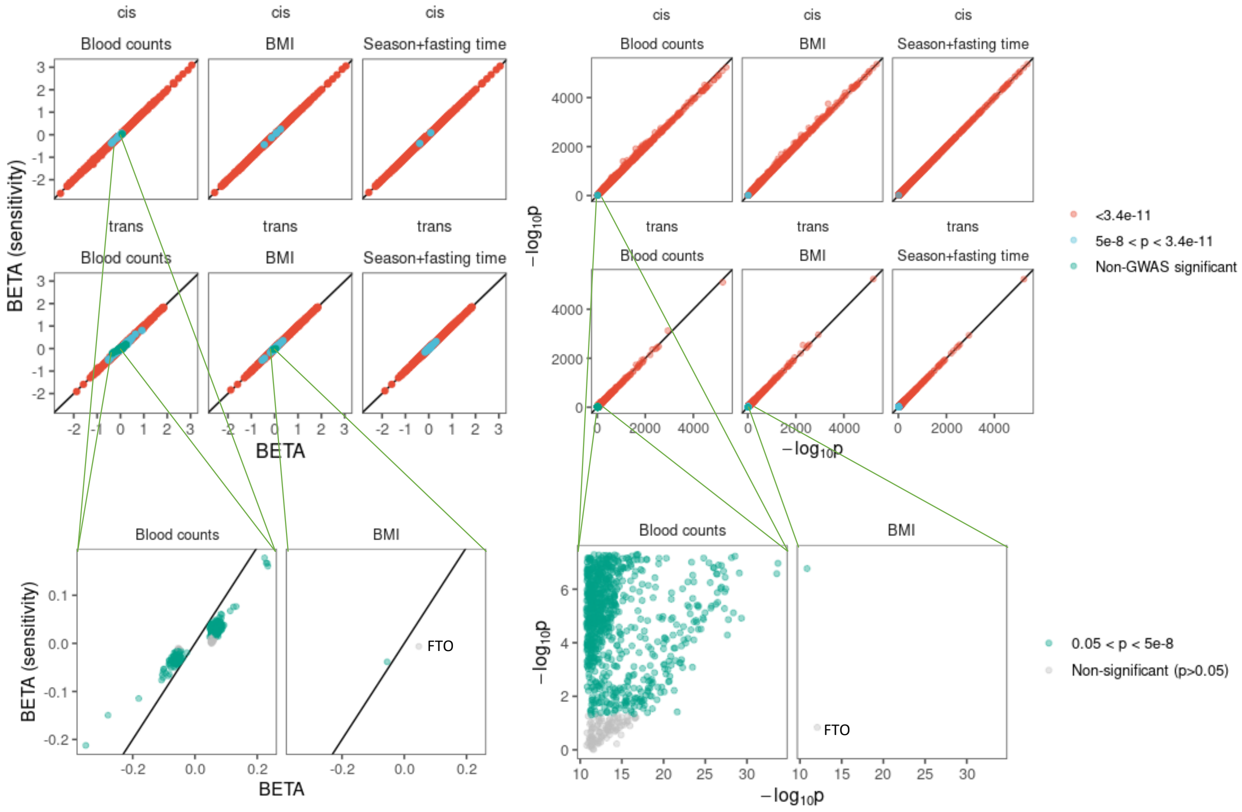

Supplementary Table 33. Proteins enriched in tissues including blood.

Assay Target	Specificity category	Enhanced tissues
CLEC1B	Group enriched	blood,liver
CLEC4C	Tissue enriched	blood
CLEC4D	Group enriched	blood,bone marrow,lymphoid tissue
CLEC6A	Group enriched	blood,heart muscle,lung,lymphoid tissue
CSF2	Tissue enhanced	blood,lung,pancreas
DRAXIN	Tissue enhanced	blood,brain
FCER2	Group enriched	blood,lymphoid tissue
FCRL1	Group enriched	blood,lymphoid tissue
FCRL2	Group enriched	blood,intestine,lymphoid tissue
FCRL3	Tissue enhanced	blood,lymphoid tissue
FCRLB	Tissue enhanced	blood,lymphoid tissue
FGF23	Tissue enhanced	blood,heart muscle,liver,urinary bladder
FOLR3	Tissue enhanced	blood,bone marrow,lymphoid tissue
GFRAL	Tissue enhanced	adipose tissue,blood,liver
HBQ1	Group enriched	blood,bone marrow,brain,vagina
IFNL1	Tissue enhanced	blood
IL27	Tissue enhanced	blood,brain,liver
KIR3DL1	Tissue enhanced	blood,bone marrow,lymphoid tissue
LAIR2	Group enriched	blood,lymphoid tissue,pituitary gland,placenta
MAP1LC3B2	Tissue enriched	blood
MCEMP1	Group enriched	blood,bone marrow,lung,lymphoid tissue
OTOA	Group enriched	blood,lymphoid tissue,testis
PADI4	Group enriched	blood,bone marrow,lymphoid tissue
PGLYRP1	Tissue enhanced	blood,bone marrow
RNASE3	Group enriched	blood,bone marrow
SCT	Group enriched	blood,intestine
SHD	Group enriched	blood,brain,heart muscle
SIGLEC5	Group enriched	blood,bone marrow
SIT1	Group enriched	blood,lymphoid tissue
TCL1A	Group enriched	blood,lymphoid tissue
TEX101	Group enriched	blood,testis
TNFRSF13B	Tissue enhanced	blood,lymphoid tissue,skeletal muscle
TNFRSF13C	Tissue enhanced	blood,intestine,lymphoid tissue
TNFRSF9	Group enriched	blood,lymphoid tissue
TNFSF11	Tissue enhanced	blood,lymphoid tissue
TREML2	Tissue enhanced	blood,bone marrow,placenta
VSTM1	Group enriched	blood,bone marrow

Figure S14. Proportion of variance explained by various *a priori* defined sensitivity factors. Each box plot presents the median, first and third quartiles, with upper and lower whiskers representing 1.5x inter-quartile range above and below the third and first quartiles respectively; n=2,940 independent protein analytes tested.

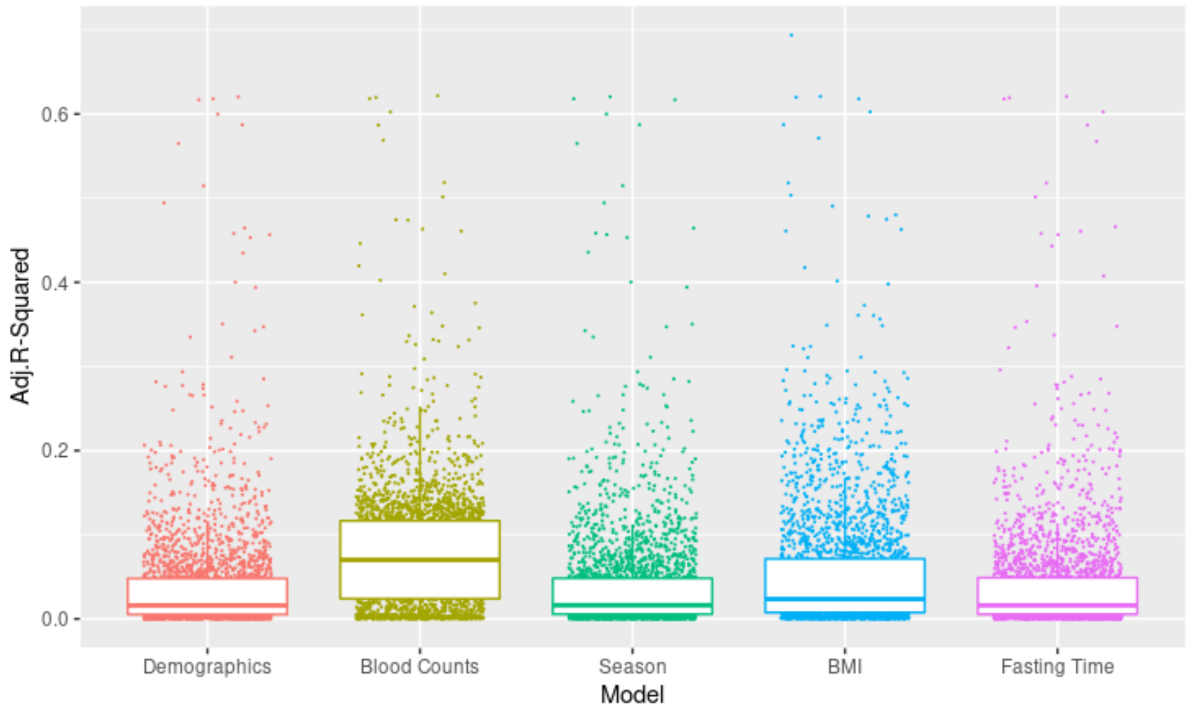

Co-localization with expression QTLs

Colocalized pairs of pQTL and eQTL signals were largely concordant with respect to their effects on circulating proteins and tissue gene expression levels, with 82% (7,728/9,385) sharing the same direction of effect overall. We observed the lowest directional concordance rates in tissues from the brain (**Extended Data Figure 10a**), including the cerebellar hemisphere (64%; 85/133) and cerebellum (68%; 100/148), and the highest in the liver (90%; 146/141), which could potentially be explained by factors affecting access to circulation such as the blood brain barrier. One example of a gene with tissue-specific discordant direction was *ADAM23*, where 5 *cis* pQTL signals colocalized with *ADAM23* eQTLs across 12 different tissues, showing discordant directions of effect for the cerebellum (**Extended Data Figure 10b**).

ABO blood group and FUT2 secretor epistasis effects provide insights into GI pathophysiology

We observed pleiotropic associations at the *ABO* blood group and fucosyltransferase 2 (*FUT2*) loci on chromosomes 9 and 19 respectively. The FUT2 enzyme facilitates expression of ABH antigens on red cells of corresponding blood groups in mucal and gastro-intestinal (GI) secretions. Approximately 20% of white Europeans are homozygous for deletion of the *FUT2* functional secretor allele (rs601338, Trp154Ter), leading to truncation and inactivation of the enzyme and non-secretion of the blood group antigens[33]. The *FUT2* deletion has been associated with cholestatic and gastrointestinal conditions[34-36]. This led us to explore the biologically informed hypothesis that FUT2 secretor status modifies the effect of blood group antigen expression on protein levels, serving as an example of long-range gene-by-gene interaction.

We found the proportion of FUT2 non-secretor carriers (25.4%) in UKB-PPP to be in line with reported prevalence and we did not observe any evidence of dependencies between *ABO* blood group genotypes and FUT2 secretor status (χ^2 p=0.58). At a multiple testing corrected threshold of $p<1.7\text{x}10^{-5}$, 432 proteins were associated with ABO blood groups and 225 proteins were associated with secretor status (**Supplementary Table 27**). We found significant interactions between blood group and secretor status for 55 proteins.

Some of the top interactions include MUC2, FAM3D and ALPI (**Figure 4a).** MUC2 levels were lower in secretors compared to non-secretors and similar between blood groups in non-secretors only, whilst in secretors, we found O blood group had lower MUC2 levels vs A and in turn AB/B blood groups (**Figure 4a left**). For FAM3D, we found higher levels in secretors, with differences between blood groups only seen in secretors where the O blood group had lower levels vs non-O, and levels were higher in A than AB and in turn B blood groups (**Figure 4a middle**). For ALPI (intestinal alkaline phosphatase), we replicated and extend on the only previous reported such interaction effect seen in a smaller targeted study for alkaline phosphatase (ALP) in a Japanese cohort[37] - we found higher levels in secretors and differences between blood groups in secretors only where O blood groups had higher levels vs non-O, and levels were lower in A than AB and in turn B blood groups (**Figure 4a right).** MUC2 is a major secretory mucin in the intestinal epithelium forming the protective mucus layer with important roles in protection against microbial GI infections[38]. FUT2 has been directly

involved in the altered glycosylation and expression of MUC2 which may be responsible for altered GI microbiome contributing to altered risk at the FUT2 locus, modulated by ABO, in Crohn's inflammatory bowel disease (IBD)[36,39,40]. FAM3D is a gut-derived protein with important roles in GI homeostasis and inflammation[41]. Notably, *FAM3D* is the strongest co-expressed RNA with *FUT2* in intestine tissues (ρ=0.95) in the Human Protein Atlas transcriptomic data[42]. This suggests FUT2 secretor effects on FAM3D in GI tissues may be tightly regulated at both transcription and protein levels. Rare bi-lateral mutations leading in *ALPI* leading to ALPI deficiency was implicated as a Mendelian cause of IBD[43]. Our results are consistent with reduced ALPI in FUT2 non-secretors which is associated with increased Crohn's IBD risk[36], and also reduced ALPI in non-O blood groups within secretors where the non-O blood group/non-secretors has been associated with Crohn's IBD risk and complications[44].

We found significant gene expression enrichments for proteins with significant interaction effects across multiple human gastrointestinal tissues[45], including duodenum, small intestine, colon, rectum, and pancreas – reinforcing with the role of FUT2 in GI secretions (**Figure 4b left)**. Enrichment in the intestine was also observed in orthologous genes in a mouse tissue expression data[46] (**Figure 4b right)**, indicating a degree of conservation between these two species.

Our results provide evidence of blood group and secretor interaction in the modulation of proteomic concentrations, which may underlie potential susceptibility mechanisms to various FUT2/ABO associated GI conditions (such as IBD) and infections.

Proteomic insights into pathways underlying COVID-19 associated loci

The COVID-19 pandemic continues to accelerate research into the mechanisms and pathways influencing risk of COVID-19 infections and potential target candidates for drug compounds. Here we integrated pQTL data with the largest GWAS meta-analysis of reported and hospitalized COVID-19 cases conducted to date (https://www.covid19hg.org/results/r7/) using multi-trait colocalization under the HyPrColoc framework[47].

For six of the COVID-19 hospitalization loci, we found high posterior probability of colocalization (PP>0.9) with pQTLs for two proteins with expression in the lungs: surfactant protein D (SFTPD) and lysosome-associated membrane glycoprotein 3 (LAMP3) (**Supplementary Table 28**). At the *MUC5B* locus, we found evidence of multi-trait colocalizations with SFTPD and LAMP3 *trans* pQTLs, driven by the *MUC5B* functional promoter variant, rs35705950 (PP=1, **Extended Data Figure 11a),** also associated with interstitial lung disease[48]. Additionally, the *cis* SFTPD association colocalized with a COVID-19 hospitalization association at the *SFTPD* locus, driven by the *SFTPD* missense variant, rs721917 (PP=0.93). SFTPD has previously been causally implicated by Mendelian randomization studies for chronic obstructive pulmonary disorder[49] and COVID-19 hospitalization[50]. SFTPD may also be indirectly targeted by viral proteins and complicate the pulmonary effects of COVID-19 leading to hospitalisation[51]. SFTPD and LAMP3 genes are strongly co-expressed with each other in the lungs (r=0.90) and in alveolar cells (r=0.73) from the Human Protein Atlas tissue RNA expression data[42]

At the *SLC22A31* COVID-19 hospitalization locus, we also found colocalizations with another *trans* LAMP3 pQTL, driven by the *SLC22A31* missense variant, rs117169628 (PP=0.998, Pro256Leu) in addition to the LAMP3 association at *MUC5B*. Both *SLC22A31* and *LAMP3* gene expression are most strongly enriched in lung alveolar epithelial cells (type 2, AT2) where *SLC22A31* is the strongest co-expressed gene with *LAMP3* (r=0.78) in single cell RNA expression data[42]. Lung AT2 cells have important roles in lung repair[52]; infection by SARS-CoV-2 leads to an intrinsic inflammatory reponse[53], and recently to induction of LAMP3 expression correlated to type I Interferon signalling[54], *in vitro*. Our results orthogonally provide additional evidence for SLC22A31 as the causative gene driving severity of COVID-19 response and hospitalizations at this locus, with the differences in risk potentially driven by differential LAMP3 expression marking inflammatory response from SARS-CoV-2 infection

within AT2 cells. We also demonstrate the LAMP3 *trans* pQTL at *SLC22A31* can identify tightly co-expressed genes within tissues/cells at the transcriptional level.

Apart from the pleiotropic *ABO* locus, all proteins showing evidence of pQTLs colocalizing with COVID-19 hospitalization loci (PP>0.7; **Supplementary Table 28)** showed a 21-fold enrichment at nominal significance for their corresponding gene expression in the lungs (p=3.6x10^{-3}).

In addition to colocalization at the pleiotropic *ABO* locus, we also found evidence of colocalization between the gene-dense region containing *TYK2*, *ICAM*-encoding genes at chromosome 19, and the interleukin-12 receptor subunit beta-1 (IL12RB1) *trans* pQTL (PP=0.97, rs34536443, *TYK2* P1104A). IL12RB1 associates with TYK2 to transmit downstream signals from IL12RB1 activation[55]. The *trans* pQTL is consistent with the causal TYK2 partial loss of function caused by P1104A and its effects in IL12R signaling as part of the immune response to COVID-19. No additional colocalizations were identified for the other proteins with *cis* associations overlapping this locus, including ICAM-1,3,4 and 5 (**Extended Data Figure 11b)**, suggesting these ICAMs are unlikely to be the risk genes for COVID-19 hospitalisations at this particular locus. Additionally, we did not observe associations with IL12RB2 (p=0.90) with the TYK2 missense variant, indicating the specificity of the proteomic effect and consistency with IL12RB2 associating with JAK2 instead of TYK2[55] (**Extended Data Figure 11c**).

The COVID-19-positive vs population case-control GWAS likely provides estimate risk of contracting COVID-19, whilst the COVID-19 hospitalisations vs population GWAS likely also provides a measure of severity of response and/or functional sequelae to COVID-19 infections. Therefore, there may be overlapping loci that influence both risk and severity (e.g. *ABO, OAS1-3*), and unique loci influencing risk only (e.g. *EFNA1, PLEKHA4*) and severity only (e.g. *TYK2, MUC5B, SFTPD*, **Supplementary Table 28)**. Additionally, we could only detect colocalizations for measured proteins covered by the assay and thus some COVID-19 loci with additional pQTL colocalizations are still yet to be detected.

Insights into inflammasome pathways

Inflammasomes are multimeric protein complexes that mediate innate immune responses, primarily through the activation of CASP1 and subsequent cleavage, activation, and non-canonical secretion of pro-inflammatory cytokines IL-18 and IL-1β[56,57]. Rare, protein altering variants in inflammasome components are known to cause many inherited autoinflammatory conditions[58]. The causal relationship between genetic alterations in the inflammasome and autoinflammation has been clinically validated by their successful treatment with anti-IL-1β therapies[59].

In this study, we observed multiple *trans* pQTL associations between inflammasome components and downstream effector proteins CASP1, IL-18, and IL-1β (**Supplementary Table 29)**. These associations included genes that encode inflammasome scaffolding proteins (*NLRC4, NLRP6*, and *NLRP12*); negative regulators of inflammasome activity (*VDR, CARD18*); and *GSDMD*, which enables the non-canonical secretion of IL-18 and IL-1β, and is an activator of pyroptosis **(Supplementary Table 29)**. These results include both previously reported *NLRP12*/IL-18 pQTLs, and novel pQTLs **(Supplementary Table 29)**.

Previous proteo-genomics studies using the SomaScan aptamer platform reported inconsistent *trans* pQTL results at the *NLRP12* locus. The INTERVAL[11] study identified this as a pleotropic hot spot, whereas AGES and a cross-platform study did not[23,60], leading to speculation that pQTL associations at this locus may be platform dependent and/or the result of inter-study differences in sample handling. The INTERVAL study identified more than 300 *trans* pQTLs within a 5Kb window of the *NLRP12* locus. Using similar filtering criteria, we identified 44 *trans* pQTLs within the same window. While head-to-head comparisons between these studies are difficult due to greater statistical power of PPP and broader protein screening of INTERVAL, these results suggest the UKB-PPP findings are more consistent with the typical pleotropic loci identified in other studies.

In contrast to other loci such as *ARHGEF3* (see Effects of blood cell counts section), *trans* pQTL results at the *NLRP12* locus were robust to cell blood count sensitivity analyses. This supports the hypotheses that these signals result from inflammasome driven biological effects, rather than artificial increases in protein concentrations caused by sample handling related cell lysis.

Furthermore, we found a missense variant in *NLRC5* (rs74439742, Pro191Gln) to be the lead *trans* pQTL associated with reduced levels of all MHC class I proteins tested: HLA-A, HLA-E and B2M, as well as BTN3A2. This is highly consistent with *NLRC5* being a key transcriptional activator of MHC class I genes[61] and recent evidence of NLRC5 promoting expression of *BTN3A1-3* genes[62]. Notably, we also found *trans* eQTLs of the missense variant for consistent, decreased expression of *HLA-E* and *BTN3A2* in the blood[63]. Pro191Gln lies within the untypical CARD domain of *NLRC5* which is predicted to be responsible for the interactions with the adaptor proteins and for the activation of downstream signaling[64] which may explain the effects observed in addition to potentially altered *NLRC5* expression. Notably, this is one of the first examples where a *trans* pQTL is also reflected at the transcriptional level as a *trans* eQTL, corroborated by consistent mechanisms of effect, suggesting this may a tightly coupled and regulated immune response pathway in MHC class I expression[65].

Taken together, these results indicate that - in addition to known, rare, highly penetrant, disease-causing variants – common forms of genetic variability play a more subtle, but significant, role in inflammasome-mediated innate immune responses.

PCSK9 MR with extended instruments expands mirroring of clinical trial pharmacological effects on cholesterol and indicated diseases

The causal effects of PCSK9 levels on LDL and total cholesterol have been well established through various orthogonal means, with several randomized clinical trials demonstrating the efficacy of PCSK9 inhibitors on cholesterol levels and cardiovascular events[66-69]. Leveraging multiple *cis* pQTLs as genetic instruments to proxy directly for the effect of PCSK9 levels, we employed Mendelian randomization to examine causal effects of PCSK9 levels on lipids (HDL, LDL and total cholesterol), cardiovascular outcomes (coronary heart disease (CHD), myocardial infarction (MI)) and ischaemic stroke (IS: large-artery (IS-LA) and small-vessel (IS-SV) subtypes) (**Methods**).

For lipids, we found significant causal effects of increased PCSK9 on increased LDL cholesterol (MR_{LDL}=0.45, p=6.1x10^{-76}) and total cholesterol (MR_{TC}=0.31, p=1.0x10^{-48}), and decreased HDL cholesterol (MR_{HDL}=-0.04, p=0.0051) (**Extended Data Figure 12a, Supplementary Table 30**). We also found significant causal associations with increased risk of CHD ($MR_{log(CHD\ OR)}$=0.23, p=1.1x10^{-9}) and MI ($MR_{log(MI\ OR)}$=0.26, p=1.9x10^{-9}). For stroke, we found significant causal associations with increased risk of large artery ischaemic stroke subtype ($MR_{log(IS\text{-}LA\ OR)}$=0.23, p=0.014). In addition to replicate previous findings on LDL, total cholesterol and CHD with even stronger MR evidence, the large sample size and increased precision in genetic instruments also point us to emerging evidence of PCSK9's effects on HDL and large artery ischaemic stroke[70,71]. The relative MR effects of PCSK9 on HDL (MR_{HDL}=-0.04) is much smaller compared to LDL (MR_{LDL}=0.45) and total cholesterol (MR_{TC}=0.31), this is in-line with the smaller effects of PCSK9 inhibition on HDL (mean difference: 6.9%), LDL (mean difference: -55%) and total cholesterol (mean difference: -35%) in randomized clinical trials[67]. Secondary mechanisms by which PCSK9 may influence HDL have been proposed which may not be directly a result of effects on LDL cholesterol[72]. These findings extend the corroborated effects observed across multiple randomised clinical trials of PCSK9 inhibitors[67]. We also compared the gain in precision for the *PCSK9* genetic instrument and MR analyses between the current study and three previous pQTL studies[73-75]. For the strongest genetic instrument in *PCSK9*, SNP rs11591147, the strength of instrument increased substantially with larger sample size in the various pQTL studies (F statistics is 17.68 in Suhre et al. 2017 [N=997] vs 2304.82 in the current study; **Supplementary Table 30**). The MR results for rs11591147 on the lipid, CHD and stroke outcomes also became more significant in the current study when

compared with instruments from previous pQTL studies, reflecting higher precision in the genetic instrument with increasing sample sizes (**Extended Data Figure 12b**). We note there is potential for a small degree of sample overlap between UKB instruments for PCSK9 and outcomes through random sampling of overlapping populations, but we expect this impact to be minimal.

UKB-PPP consortium banner contributors

Institute	Name	Address
Alnylam Human Genetics	Aimee M Deaton	Alnylam Human Genetics, Discovery & Translational Research, Alnylam Pharmaceuticals Cambridge, MA US
Alnylam Human Genetics	Rachel Hoffing	Alnylam Human Genetics, Discovery & Translational Research, Alnylam Pharmaceuticals Cambridge, MA US
Alnylam Human Genetics	Aaron M Holleman	Alnylam Human Genetics, Discovery & Translational Research, Alnylam Pharmaceuticals Cambridge, MA US
Alnylam Human Genetics	Lynne Krohn	Alnylam Human Genetics, Discovery & Translational Research, Alnylam Pharmaceuticals Cambridge, MA US
Alnylam Human Genetics	Philip LoGerfo	Alnylam Human Genetics, Discovery & Translational Research, Alnylam Pharmaceuticals Cambridge, MA US
Alnylam Human Genetics	Paul Nioi	Alnylam Human Genetics, Discovery & Translational Research, Alnylam Pharmaceuticals Cambridge, MA US
Alnylam Human Genetics	Mollie E Plekan	Alnylam Human Genetics, Discovery & Translational Research, Alnylam Pharmaceuticals Cambridge, MA US
Alnylam Human Genetics	Lucas D Ward	Alnylam Human Genetics, Discovery & Translational Research, Alnylam Pharmaceuticals Cambridge, MA US
Alnylam Human Genetics	Carissa Willis	Alnylam Human Genetics, Discovery & Translational Research, Alnylam Pharmaceuticals Cambridge, MA US
AstraZeneca Genomics Initiative	Bastian Angermann	Translational Science and Experimental Medicine, Research and Early Development, Respiratory and Immunology, BioPharmaceuticals R&D, AstraZeneca Gothenburg Sweden
AstraZeneca Genomics Initiative	Oliver Burren	Centre for Genomics Research, Discovery Sciences, BioPharmaceuticals R&D, AstraZeneca Cambridge UK
AstraZeneca Genomics Initiative	Keren Carss	Centre for Genomics Research, Discovery Sciences, BioPharmaceuticals R&D, AstraZeneca Cambridge UK
AstraZeneca Genomics Initiative	Ryan Dhindsa	Centre for Genomics Research, Discovery Sciences, BioPharmaceuticals R&D, AstraZeneca Cambridge USA
AstraZeneca Genomics Initiative	Ian Henry	Translational Science & Experimental Medicine, Research and Early Development, Cardiovascular, Renal and Metabolism, BioPharmaceuticals R&D, AstraZeneca Cambridge UK
AstraZeneca Genomics Initiative	Ventzi Hristova	Centre for Genomics Research, Discovery Sciences, BioPharmaceuticals R&D, AstraZeneca Gaithersburg USA
AstraZeneca Genomics Initiative	Daniel Muthas	Translational Science and Experimental Medicine, Research and Early Development, Respiratory and Immunology, BioPharmaceuticals R&D, AstraZeneca Gothenburg Sweden
AstraZeneca Genomics Initiative	Erin Oerton	Centre for Genomics Research, Discovery Sciences, BioPharmaceuticals R&D, AstraZeneca Cambridge UK
AstraZeneca Genomics Initiative	Menelas N Pangalos	BioPharmaceuticals R&D, AstraZeneca Cambridge UK
AstraZeneca Genomics Initiative	Dirk Paul	Centre for Genomics Research, Discovery Sciences, BioPharmaceuticals R&D, AstraZeneca Cambridge UK
AstraZeneca Genomics Initiative	Slave Petrovski	Centre for Genomics Research, Discovery Sciences, BioPharmaceuticals R&D, AstraZeneca Cambridge UK
AstraZeneca Genomics Initiative	Adam Platt	Translational Science and Experimental Medicine, Research and Early Development, Respiratory and Immunology, BioPharmaceuticals R&D, AstraZeneca Cambridge UK
AstraZeneca Genomics Initiative	Bram Prins	Centre for Genomics Research, Discovery Sciences, BioPharmaceuticals R&D, AstraZeneca Cambridge UK
AstraZeneca Genomics Initiative	Ben Sidders	Bioinformatics and Data Science, Research and Early Development, Oncology R&D, AstraZeneca Cambridge UK
AstraZeneca Genomics Initiative	Katherine R Smith	Centre for Genomics Research, Discovery Sciences, BioPharmaceuticals R&D, AstraZeneca Cambridge UK
AstraZeneca Genomics Initiative	Quanli Wang	Centre for Genomics Research, Discovery Sciences, BioPharmaceuticals R&D, AstraZeneca Cambridge USA
AstraZeneca Genomics Initiative	Sebastian Wasilewski	Centre for Genomics Research, Discovery Sciences, BioPharmaceuticals R&D, AstraZeneca Cambridge UK
AstraZeneca Genomics Initiative	Eleanor Wheeler	Centre for Genomics Research, Discovery Sciences, BioPharmaceuticals R&D, AstraZeneca Cambridge UK
Biogen Biobank Team	Denis Baird	Research & Development, Biogen Inc. Cambridge, MA US
Biogen Biobank Team	Paola Bronson	Research & Development, Biogen Inc. Cambridge, MA US
Biogen Biobank Team	Danai Chasioti	Research & Development, Biogen Inc. Cambridge, MA US
Biogen Biobank Team	Chia-Yen Chen	Research & Development, Biogen Inc. Cambridge, MA US
Biogen Biobank Team	Susan Eaton	Research & Development, Biogen Inc. Cambridge, MA US
Biogen Biobank Team	Amanda Edwards	Research & Development, Biogen Inc. Cambridge, MA US
Biogen Biobank Team	Kyle Ferber	Research & Development, Biogen Inc. Cambridge, MA US
Biogen Biobank Team	Jake Gagnon	Research & Development, Biogen Inc. Cambridge, MA US
Biogen Biobank Team	Feng Gao	Research & Development, Biogen Inc. Cambridge, MA US
Biogen Biobank Team	Cynthia Gubbels	Research & Development, Biogen Inc. Cambridge, MA US
Biogen Biobank Team	Yunfeng Huang	Research & Development, Biogen Inc. Cambridge, MA US
Biogen Biobank Team	Megan Jensen	Research & Development, Biogen Inc. Cambridge, MA US
Biogen Biobank Team	Sally John	Research & Development, Biogen Inc. Cambridge, MA US
Biogen Biobank Team	Varant Kupelian	Research & Development, Biogen Inc. Cambridge, MA US
Biogen Biobank Team	Kejie Li	Research & Development, Biogen Inc. Cambridge, MA US
Biogen Biobank Team	Stephanie Loomis	Research & Development, Biogen Inc. Cambridge, MA US
Biogen Biobank Team	Eric Marshall	Research & Development, Biogen Inc. Cambridge, MA US
Biogen Biobank Team	Helen McLaughlin	Research & Development, Biogen Inc. Cambridge, MA US
Biogen Biobank Team	Adele Mitchell	Research & Development, Biogen Inc. Cambridge, MA US
Biogen Biobank Team	Mehool Patel	Research & Development, Biogen Inc. Cambridge, MA US
Biogen Biobank Team	Heiko Runz	Research & Development, Biogen Inc. Cambridge, MA US
Biogen Biobank Team	Benjamin B Sun	Research & Development, Biogen Inc. Cambridge, MA US
Biogen Biobank Team	Ellen Tsai	Research & Development, Biogen Inc. Cambridge, MA US
Biogen Biobank Team	Christopher D Whelan	Research & Development, Biogen Inc. Cambridge, MA US
Bristol Myers Squibb	Ian Catlett	Research and Early Development, BMS Princeton, NJ US
Bristol Myers Squibb	Ashok Dongre	Research and Early Development, BMS Cambridge, MA US
Bristol Myers Squibb	Joseph Maranville	Research and Early Development, BMS Cambridge, MA US
Bristol Myers Squibb	Peter Schafer	Research and Early Development, BMS Princeton, NJ US
Bristol Myers Squibb	Tai Wang	Research and Early Development, BMS Cambridge, MA US
Calico Life Sciences	Madeleine Cule	Computing and Data Science, Calico Life Sciences, LLC South San Francisco, CA US

Calico Life Sciences	Eugene Melamud	Computational Biology, Calico Life Sciences, LLC South San Francisco, CA US
Calico Life Sciences	Anil Raj	Computing and Data Science, Calico Life Sciences, LLC South San Francisco, CA US
Calico Life Sciences	Anurag Sethi	Computational Biology, Calico Life Sciences, LLC South San Francisco, CA US
Genentech Human Genetics	Christian Benner	Genentech South San Francisco, CA US
Genentech Human Genetics	Jerome Irudayanathan	Genentech South San Francisco, CA US
Genentech Human Genetics	Anubha Mahajan	Genentech South San Francisco, CA US
Genentech Human Genetics	Mark I McCarthy	Genentech South San Francisco, CA US
Genentech Human Genetics	Rion K Pendergrass	Genentech South San Francisco, CA US
Genentech Human Genetics	Xiaoman Xie	Genentech South San Francisco, CA US
GlaxoSmithKline Genomic Sciences	Elena Arciero	Genomic Sciences, GlaxoSmithKline Stevenage, UK
GlaxoSmithKline Genomic Sciences	Joanna C. Betts	Genomic Sciences, GlaxoSmithKline Stevenage, UK
GlaxoSmithKline Genomic Sciences	Nicholas Bowker	Genomic Sciences, GlaxoSmithKline Stevenage, UK
GlaxoSmithKline Genomic Sciences	Audrey Y. Chu	Genomic Sciences, GlaxoSmithKline Collegeville, PA US
GlaxoSmithKline Genomic Sciences	Adrian Cortes	Genomic Sciences, GlaxoSmithKline Stevenage, UK
GlaxoSmithKline Genomic Sciences	Damien C. Croteau-Chonka	Genomic Sciences, GlaxoSmithKline Collegeville, PA US
GlaxoSmithKline Genomic Sciences	Emmanouil T. Dermitzakis	Genomic Sciences, GlaxoSmithKline Collegeville, PA US
GlaxoSmithKline Genomic Sciences	Margaret G. Ehm	Genomic Sciences, GlaxoSmithKline Collegeville, PA US
GlaxoSmithKline Genomic Sciences	Stephan Gade	Genomic Sciences, GlaxoSmithKline Heidelberg Germany
GlaxoSmithKline Genomic Sciences	Padhraig Gormley	Genomic Sciences, GlaxoSmithKline Collegeville, PA US
GlaxoSmithKline Genomic Sciences	Erik D. Ingelsson	Genomic Sciences, GlaxoSmithKline Collegeville, PA US
GlaxoSmithKline Genomic Sciences	Toby Johnson	Genomic Sciences, GlaxoSmithKline Stevenage, UK
GlaxoSmithKline Genomic Sciences	Jimmy Zhenli Liu	Genomic Sciences, GlaxoSmithKline Collegeville, PA US
GlaxoSmithKline Genomic Sciences	Yancy Lo	Genomic Sciences, GlaxoSmithKline Collegeville, PA US
GlaxoSmithKline Genomic Sciences	Jatin Sandhuria	Genomic Sciences, GlaxoSmithKline Stevenage, UK
GlaxoSmithKline Genomic Sciences	Richard M. Turner	Genomic Sciences, GlaxoSmithKline Stevenage, UK
GlaxoSmithKline Genomic Sciences	Qin Wang	Genomic Sciences, GlaxoSmithKline Stevenage, UK
GlaxoSmithKline Genomic Sciences	Frederik Ziebell	Genomic Sciences, GlaxoSmithKline Heidelberg Germany
Pfizer Integrative Biology	Xinli Hu	Inflammation and Immunology Research Unit, Worldwide Research, Development and Medical, Pfizer Cambridge, MA US
Pfizer Integrative Biology	Craig L. Hyde	Non-Clinical Research Statistics, Early Clinical Development, Worldwide Research, Development and Medical, Pfizer Groton, CT US
Pfizer Integrative Biology	Hye In Kim	Internal Medicine Research Unit, Worldwide Research, Development and Medical, Pfizer Cambridge, MA US
Pfizer Integrative Biology	A. Katrina Loomis	External Science and Innovation Target Sciences, Worldwide Research, Development and Medical, Pfizer Groton, CT US
Pfizer Integrative Biology	Anders Malarstig	External Science and Innovation Target Sciences, Worldwide Research, Development and Medical, Pfizer Solletuna Sweden
Pfizer Integrative Biology	Zhan Ye	Non-Clinical Research Statistics, Early Clinical Development, Worldwide Research, Development and Medical, Pfizer Cambridge, MA US
AI/ML and DH, Data Science & Digital Health, Janssen R&D	Gamal Abdel-Azim	AI/ML and DH, Data Science & Digital Health, Janssen R&D Spring House, PA US
AI/ML and DH, Data Science & Digital Health, Janssen R&D	Evan H Baugh	AI/ML and DH, Data Science & Digital Health, Janssen R&D Spring House, PA US
AI/ML and DH, Data Science & Digital Health, Janssen R&D	Mary Helen Black	AI/ML and DH, Data Science & Digital Health, Janssen R&D Spring House, PA US
AI/ML and DH, Data Science & Digital Health, Janssen R&D	Abolfazl Doostparast Torshizi	AI/ML and DH, Data Science & Digital Health, Janssen R&D Spring House, PA US
AI/ML and DH, Data Science & Digital Health, Janssen R&D	Shicheng Guo	AI/ML and DH, Data Science & Digital Health, Janssen R&D Spring House, PA US
AI/ML and DH, Data Science & Digital Health, Janssen R&D	Karen Y He	AI/ML and DH, Data Science & Digital Health, Janssen R&D Spring House, PA US
AI/ML and DH, Data Science & Digital Health, Janssen R&D	Hussein A Hejase	AI/ML and DH, Data Science & Digital Health, Janssen R&D Spring House, PA US
AI/ML and DH, Data Science & Digital Health, Janssen R&D	Liping Hou	AI/ML and DH, Data Science & Digital Health, Janssen R&D Spring House, PA US
AI/ML and DH, Data Science & Digital Health, Janssen R&D	Ekaterina A Khramtsova	AI/ML and DH, Data Science & Digital Health, Janssen R&D Spring House, PA US
AI/ML and DH, Data Science & Digital Health, Janssen R&D	Alexander H Li	AI/ML and DH, Data Science & Digital Health, Janssen R&D Spring House, PA US
AI/ML and DH, Data Science & Digital Health, Janssen R&D	Shuwei Li	AI/ML and DH, Data Science & Digital Health, Janssen R&D Spring House, PA US
AI/ML and DH, Data Science & Digital Health, Janssen R&D	Xingjun Liu	AI/ML and DH, Data Science & Digital Health, Janssen R&D Spring House, PA US
AI/ML and DH, Data Science & Digital Health, Janssen R&D	Tommaso Mansi	AI/ML and DH, Data Science & Digital Health, Janssen R&D Titusville, NJ US
AI/ML and DH, Data Science & Digital Health, Janssen R&D	Brian Mautz	AI/ML and DH, Data Science & Digital Health, Janssen R&D Spring House, PA US
AI/ML and DH, Data Science & Digital Health, Janssen R&D	Elisabeth Mlynarski	AI/ML and DH, Data Science & Digital Health, Janssen R&D Spring House, PA US

AI/ML and DH, Data Science & Digital Health, Janssen R&D	Julio Molineros	AI/ML and DH, Data Science & Digital Health, Janssen R&D Spring House, PA US
AI/ML and DH, Data Science & Digital Health, Janssen R&D	Antonio R Parrado	AI/ML and DH, Data Science & Digital Health, Janssen R&D Spring House, PA US
AI/ML and DH, Data Science & Digital Health, Janssen R&D	Parth Patel	AI/ML and DH, Data Science & Digital Health, Janssen R&D Spring House, PA US
AI/ML and DH, Data Science & Digital Health, Janssen R&D	Brice AJ Sarver	AI/ML and DH, Data Science & Digital Health, Janssen R&D Spring House, PA US
AI/ML and DH, Data Science & Digital Health, Janssen R&D	Dongnhu Truong	AI/ML and DH, Data Science & Digital Health, Janssen R&D Spring House, PA US
AI/ML and DH, Data Science & Digital Health, Janssen R&D	Yanfei Zhang	AI/ML and DH, Data Science & Digital Health, Janssen R&D Spring House, PA US
Regeneron Genetics Center	Gonçalo Abecasis	Regeneron Genetics Center, Tarrytown, NY, US
Regeneron Genetics Center	Parsa Akbari	Regeneron Genetics Center, Tarrytown, NY, US
Regeneron Genetics Center	Anna Alkelai	Regeneron Genetics Center, Tarrytown, NY, US
Regeneron Genetics Center	Manuel Allen Revez Ferreira	Regeneron Genetics Center, Tarrytown, NY, US
Regeneron Genetics Center	Silvia Alvarez	Regeneron Genetics Center, Tarrytown, NY, US
Regeneron Genetics Center	Amelia Averitt	Regeneron Genetics Center, Tarrytown, NY, US
Regeneron Genetics Center	Ariane Ayer	Regeneron Genetics Center, Tarrytown, NY, US
Regeneron Genetics Center	Joshua Backman	Regeneron Genetics Center, Tarrytown, NY, US
Regeneron Genetics Center	Xiaodong Bai	Regeneron Genetics Center, Tarrytown, NY, US
Regeneron Genetics Center	Suganthi Balasubramanian	Regeneron Genetics Center, Tarrytown, NY, US
Regeneron Genetics Center	Antoine Baldassari	Regeneron Genetics Center, Tarrytown, NY, US
Regeneron Genetics Center	Nilanjana Banerjee	Regeneron Genetics Center, Tarrytown, NY, US
Regeneron Genetics Center	Suying Bao	Regeneron Genetics Center, Tarrytown, NY, US
Regeneron Genetics Center	Aris Baras	Regeneron Genetics Center, Tarrytown, NY, US
Regeneron Genetics Center	Christina Beechert	Regeneron Genetics Center, Tarrytown, NY, US
Regeneron Genetics Center	Boris Boutkov	Regeneron Genetics Center, Tarrytown, NY, US
Regeneron Genetics Center	Jonas Bovijn	Regeneron Genetics Center, Tarrytown, NY, US
Regeneron Genetics Center	Erin D Brian	Regeneron Genetics Center, Tarrytown, NY, US
Regeneron Genetics Center	Jessie Brown	Regeneron Genetics Center, Tarrytown, NY, US
Regeneron Genetics Center	Andrew Bunyea	Regeneron Genetics Center, Tarrytown, NY, US
Regeneron Genetics Center	Kathy Burch	Regeneron Genetics Center, Tarrytown, NY, US
Regeneron Genetics Center	Adrian Campos	Regeneron Genetics Center, Tarrytown, NY, US
Regeneron Genetics Center	Michael Cantor	Regeneron Genetics Center, Tarrytown, NY, US
Regeneron Genetics Center	Lei Chen	Regeneron Genetics Center, Tarrytown, NY, US
Regeneron Genetics Center	Esteban Chen	Regeneron Genetics Center, Tarrytown, NY, US
Regeneron Genetics Center	Sam Choi	Regeneron Genetics Center, Tarrytown, NY, US
Regeneron Genetics Center	Janice Clauer	Regeneron Genetics Center, Tarrytown, NY, US
Regeneron Genetics Center	Thomas Coleman	Regeneron Genetics Center, Tarrytown, NY, US
Regeneron Genetics Center	Giovanni Coppola	Regeneron Genetics Center, Tarrytown, NY, US
Regeneron Genetics Center	Ruan Cox	Regeneron Genetics Center, Tarrytown, NY, US
Regeneron Genetics Center	Laura M Cremona	Regeneron Genetics Center, Tarrytown, NY, US
Regeneron Genetics Center	Amy Damask	Regeneron Genetics Center, Tarrytown, NY, US
Regeneron Genetics Center	Tanima De	Regeneron Genetics Center, Tarrytown, NY, US
Regeneron Genetics Center	Andrew Deubler	Regeneron Genetics Center, Tarrytown, NY, US
Regeneron Genetics Center	Lee Dobbyn	Regeneron Genetics Center, Tarrytown, NY, US
Regeneron Genetics Center	Peter Dombos	Regeneron Genetics Center, Tarrytown, NY, US
Regeneron Genetics Center	Hang Du	Regeneron Genetics Center, Tarrytown, NY, US
Regeneron Genetics Center	Aris Economides	Regeneron Genetics Center, Tarrytown, NY, US
Regeneron Genetics Center	Evan Edelstein	Regeneron Genetics Center, Tarrytown, NY, US
Regeneron Genetics Center	Gisu Eom	Regeneron Genetics Center, Tarrytown, NY, US
Regeneron Genetics Center	Alison Fenney	Regeneron Genetics Center, Tarrytown, NY, US
Regeneron Genetics Center	Adolfo Ferrando	Regeneron Genetics Center, Tarrytown, NY, US
Regeneron Genetics Center	Caitlin Forsythe	Regeneron Genetics Center, Tarrytown, NY, US
Regeneron Genetics Center	Jan Freudenberg	Regeneron Genetics Center, Tarrytown, NY, US
Regeneron Genetics Center	Liron Ganel	Regeneron Genetics Center, Tarrytown, NY, US
Regeneron Genetics Center	Sheila Gaynor	Regeneron Genetics Center, Tarrytown, NY, US
Regeneron Genetics Center	Sahar Gelfman	Regeneron Genetics Center, Tarrytown, NY, US
Regeneron Genetics Center	Benjamin Geraghty	Regeneron Genetics Center, Tarrytown, NY, US
Regeneron Genetics Center	Akropravo Ghosh	Regeneron Genetics Center, Tarrytown, NY, US
Regeneron Genetics Center	Christopher Gillies	Regeneron Genetics Center, Tarrytown, NY, US
Regeneron Genetics Center	Arthur Gilly	Regeneron Genetics Center, Tarrytown, NY, US
Regeneron Genetics Center	Sujit Gokhale	Regeneron Genetics Center, Tarrytown, NY, US
Regeneron Genetics Center	Alexander Gorovits	Regeneron Genetics Center, Tarrytown, NY, US
Regeneron Genetics Center	Sarah Graham	Regeneron Genetics Center, Tarrytown, NY, US
Regeneron Genetics Center	Zhenhua Gu	Regeneron Genetics Center, Tarrytown, NY, US
Regeneron Genetics Center	Ju Guan	Regeneron Genetics Center, Tarrytown, NY, US
Regeneron Genetics Center	Kristy Guevara	Regeneron Genetics Center, Tarrytown, NY, US
Regeneron Genetics Center	Lauren Gurski	Regeneron Genetics Center, Tarrytown, NY, US
Regeneron Genetics Center	Aysegul Guvenek	Regeneron Genetics Center, Tarrytown, NY, US
Regeneron Genetics Center	Mary Haas	Regeneron Genetics Center, Tarrytown, NY, US
Regeneron Genetics Center	Lukas Habegger	Regeneron Genetics Center, Tarrytown, NY, US
Regeneron Genetics Center	Jody Hankins	Regeneron Genetics Center, Tarrytown, NY, US
Regeneron Genetics Center	Samuel Hart	Regeneron Genetics Center, Tarrytown, NY, US
Regeneron Genetics Center	Alicia Hawes	Regeneron Genetics Center, Tarrytown, NY, US
Regeneron Genetics Center	Jin He	Regeneron Genetics Center, Tarrytown, NY, US
Regeneron Genetics Center	Joseph Herman	Regeneron Genetics Center, Tarrytown, NY, US
Regeneron Genetics Center	Jaimee Hernandez	Regeneron Genetics Center, Tarrytown, NY, US
Regeneron Genetics Center	George Hindy	Regeneron Genetics Center, Tarrytown, NY, US
Regeneron Genetics Center	Brian Hobbs	Regeneron Genetics Center, Tarrytown, NY, US
Regeneron Genetics Center	Marcus B Jones	Regeneron Genetics Center, Tarrytown, NY, US
Regeneron Genetics Center	Eric Jorgenson	Regeneron Genetics Center, Tarrytown, NY, US
Regeneron Genetics Center	Tyler Joseph	Regeneron Genetics Center, Tarrytown, NY, US
Regeneron Genetics Center	Amit Joshi	Regeneron Genetics Center, Tarrytown, NY, US
Regeneron Genetics Center	Manav Kapoor	Regeneron Genetics Center, Tarrytown, NY, US
Regeneron Genetics Center	Katia Karalis	Regeneron Genetics Center, Tarrytown, NY, US

Regeneron Genetics Center	Michael Kessler	Regeneron Genetics Center, Tarrytown, NY, US
Regeneron Genetics Center	Hossein Khiabanian	Regeneron Genetics Center, Tarrytown, NY, US
Regeneron Genetics Center	Jack Kosmicki	Regeneron Genetics Center, Tarrytown, NY, US
Regeneron Genetics Center	Olga Krasheninina	Regeneron Genetics Center, Tarrytown, NY, US
Regeneron Genetics Center	Vijay Kumar	Regeneron Genetics Center, Tarrytown, NY, US
Regeneron Genetics Center	Rouel Lanche	Regeneron Genetics Center, Tarrytown, NY, US
Regeneron Genetics Center	Michael Lattari	Regeneron Genetics Center, Tarrytown, NY, US
Regeneron Genetics Center	Michelle G LeBlanc	Regeneron Genetics Center, Tarrytown, NY, US
Regeneron Genetics Center	Dadong Li	Regeneron Genetics Center, Tarrytown, NY, US
Regeneron Genetics Center	Nan Lin	Regeneron Genetics Center, Tarrytown, NY, US
Regeneron Genetics Center	Adam Locke	Regeneron Genetics Center, Tarrytown, NY, US
Regeneron Genetics Center	Alexander Lopez	Regeneron Genetics Center, Tarrytown, NY, US
Regeneron Genetics Center	Luca A Lotta	Regeneron Genetics Center, Tarrytown, NY, US
Regeneron Genetics Center	Vrushali Mahajan	Regeneron Genetics Center, Tarrytown, NY, US
Regeneron Genetics Center	Koteswararao Makkena	Regeneron Genetics Center, Tarrytown, NY, US
Regeneron Genetics Center	Sameer Malhotra	Regeneron Genetics Center, Tarrytown, NY, US
Regeneron Genetics Center	Kia Manoochehri	Regeneron Genetics Center, Tarrytown, NY, US
Regeneron Genetics Center	Adam J Mansfield	Regeneron Genetics Center, Tarrytown, NY, US
Regeneron Genetics Center	Jonathan Marchini	Regeneron Genetics Center, Tarrytown, NY, US
Regeneron Genetics Center	Anthony Marcketta	Regeneron Genetics Center, Tarrytown, NY, US
Regeneron Genetics Center	Evan K Maxwell	Regeneron Genetics Center, Tarrytown, NY, US
Regeneron Genetics Center	Joelle Mbatchou	Regeneron Genetics Center, Tarrytown, NY, US
Regeneron Genetics Center	Jason Mighty	Regeneron Genetics Center, Tarrytown, NY, US
Regeneron Genetics Center	Lyndon J Mitnaul	Regeneron Genetics Center, Tarrytown, NY, US
Regeneron Genetics Center	George Mitra	Regeneron Genetics Center, Tarrytown, NY, US
Regeneron Genetics Center	Arden Moscati	Regeneron Genetics Center, Tarrytown, NY, US
Regeneron Genetics Center	Justin Mower	Regeneron Genetics Center, Tarrytown, NY, US
Regeneron Genetics Center	Mona Nafde	Regeneron Genetics Center, Tarrytown, NY, US
Regeneron Genetics Center	Priyanka Nakka	Regeneron Genetics Center, Tarrytown, NY, US
Regeneron Genetics Center	Nirupama Nishtala	Regeneron Genetics Center, Tarrytown, NY, US
Regeneron Genetics Center	Sean O'Keeffe	Regeneron Genetics Center, Tarrytown, NY, US
Regeneron Genetics Center	Jacqueline Otto	Regeneron Genetics Center, Tarrytown, NY, US
Regeneron Genetics Center	John D Overton	Regeneron Genetics Center, Tarrytown, NY, US
Regeneron Genetics Center	Billy Palmer	Regeneron Genetics Center, Tarrytown, NY, US
Regeneron Genetics Center	Aditeya Pandey	Regeneron Genetics Center, Tarrytown, NY, US
Regeneron Genetics Center	Anita Pandit	Regeneron Genetics Center, Tarrytown, NY, US
Regeneron Genetics Center	Razvan Panea	Regeneron Genetics Center, Tarrytown, NY, US
Regeneron Genetics Center	Neel Parikshak	Regeneron Genetics Center, Tarrytown, NY, US
Regeneron Genetics Center	Charles Paulding	Regeneron Genetics Center, Tarrytown, NY, US
Regeneron Genetics Center	Krishna Pawan Punuru	Regeneron Genetics Center, Tarrytown, NY, US
Regeneron Genetics Center	Tommy Polanco	Regeneron Genetics Center, Tarrytown, NY, US
Regeneron Genetics Center	Manasi Pradhan	Regeneron Genetics Center, Tarrytown, NY, US
Regeneron Genetics Center	Kavita Praveen	Regeneron Genetics Center, Tarrytown, NY, US
Regeneron Genetics Center	Veera Rajagopal	Regeneron Genetics Center, Tarrytown, NY, US
Regeneron Genetics Center	Nadia Rana	Regeneron Genetics Center, Tarrytown, NY, US
Regeneron Genetics Center	Ayesha Rasool	Regeneron Genetics Center, Tarrytown, NY, US
Regeneron Genetics Center	Jeffrey G Reid	Regeneron Genetics Center, Tarrytown, NY, US
Regeneron Genetics Center	Raymond Reynoso	Regeneron Genetics Center, Tarrytown, NY, US
Regeneron Genetics Center	Moeen Riaz	Regeneron Genetics Center, Tarrytown, NY, US
Regeneron Genetics Center	Jennifer Rico-Varela	Regeneron Genetics Center, Tarrytown, NY, US
Regeneron Genetics Center	Juan Rodriguez-Flores	Regeneron Genetics Center, Tarrytown, NY, US
Regeneron Genetics Center	Jonathan Ross	Regeneron Genetics Center, Tarrytown, NY, US
Regeneron Genetics Center	William J Salerno	Regeneron Genetics Center, Tarrytown, NY, US
Regeneron Genetics Center	Mudasar Sarwar	Regeneron Genetics Center, Tarrytown, NY, US
Regeneron Genetics Center	Ricardo Schiavo	Regeneron Genetics Center, Tarrytown, NY, US
Regeneron Genetics Center	Randi Schwartz	Regeneron Genetics Center, Tarrytown, NY, US
Regeneron Genetics Center	Deepika Sharma	Regeneron Genetics Center, Tarrytown, NY, US
Regeneron Genetics Center	Alan Shuldiner	Regeneron Genetics Center, Tarrytown, NY, US
Regeneron Genetics Center	Carlo Sidore	Regeneron Genetics Center, Tarrytown, NY, US
Regeneron Genetics Center	Katherine Siminovitch	Regeneron Genetics Center, Tarrytown, NY, US
Regeneron Genetics Center	Olukayode Sosina	Regeneron Genetics Center, Tarrytown, NY, US
Regeneron Genetics Center	Kayode Sosina	Regeneron Genetics Center, Tarrytown, NY, US
Regeneron Genetics Center	Maria Sotiropoulos Padilla	Regeneron Genetics Center, Tarrytown, NY, US
Regeneron Genetics Center	Sanjay Sreeram	Regeneron Genetics Center, Tarrytown, NY, US
Regeneron Genetics Center	Eli Stahl	Regeneron Genetics Center, Tarrytown, NY, US
Regeneron Genetics Center	Jeffrey C Staples	Regeneron Genetics Center, Tarrytown, NY, US
Regeneron Genetics Center	Maria Suciu	Regeneron Genetics Center, Tarrytown, NY, US
Regeneron Genetics Center	Benjamin Sultan	Regeneron Genetics Center, Tarrytown, NY, US
Regeneron Genetics Center	Kathie Sun	Regeneron Genetics Center, Tarrytown, NY, US
Regeneron Genetics Center	Luanluan Sun	Regeneron Genetics Center, Tarrytown, NY, US
Regeneron Genetics Center	Jay Sundaram	Regeneron Genetics Center, Tarrytown, NY, US
Regeneron Genetics Center	Timothy Thornton	Regeneron Genetics Center, Tarrytown, NY, US
Regeneron Genetics Center	Gannie Tzoneva	Regeneron Genetics Center, Tarrytown, NY, US
Regeneron Genetics Center	Peter VandeHaar	Regeneron Genetics Center, Tarrytown, NY, US
Regeneron Genetics Center	Sailaja Vedantam	Regeneron Genetics Center, Tarrytown, NY, US
Regeneron Genetics Center	Niek Verweij	Regeneron Genetics Center, Tarrytown, NY, US
Regeneron Genetics Center	Scott Vrieze	Regeneron Genetics Center, Tarrytown, NY, US
Regeneron Genetics Center	Rujin Wang	Regeneron Genetics Center, Tarrytown, NY, US
Regeneron Genetics Center	Chenggu Wang	Regeneron Genetics Center, Tarrytown, NY, US
Regeneron Genetics Center	Kyoko Watanabe	Regeneron Genetics Center, Tarrytown, NY, US
Regeneron Genetics Center	Cristen J Willer	Regeneron Genetics Center, Tarrytown, NY, US
Regeneron Genetics Center	Sarah E Wolf	Regeneron Genetics Center, Tarrytown, NY, US
Regeneron Genetics Center	Kuan-Han Wu	Regeneron Genetics Center, Tarrytown, NY, US
Regeneron Genetics Center	Bin Ye	Regeneron Genetics Center, Tarrytown, NY, US
Regeneron Genetics Center	Sean Yu	Regeneron Genetics Center, Tarrytown, NY, US
Regeneron Genetics Center	Blair Zhang	Regeneron Genetics Center, Tarrytown, NY, US
Regeneron Genetics Center	Aaron Zhang	Regeneron Genetics Center, Tarrytown, NY, US

Regeneron Genetics Center	Lance Zhang	Regeneron Genetics Center, Tarrytown, NY, US
Regeneron Genetics Center	Andrey Ziyatdinov	Regeneron Genetics Center, Tarrytown, NY, US
Regeneron Genetics Center	Yuxin Zou	Regeneron Genetics Center, Tarrytown, NY, US

References

1 Allen, N. E. *et al.* Approaches to minimising the epidemiological impact of sources of systematic and random variation that may affect biochemistry assay data in UK Biobank. *Wellcome Open Res* **5**, 222, doi:10.12688/wellcomeopenres.16171.2 (2020).

2 Elliott, P., Peakman, T. C. & Biobank, U. K. The UK Biobank sample handling and storage protocol for the collection, processing and archiving of human blood and urine. *Int J Epidemiol* **37**, 234-244, doi:10.1093/ije/dym276 (2008).

3 Wik, L. *et al.* Proximity Extension Assay in Combination with Next-Generation Sequencing for High-throughput Proteome-wide Analysis. *Mol Cell Proteomics* **20**, 100168, doi:10.1016/j.mcpro.2021.100168 (2021).

4 Zhong, W. *et al.* Next generation plasma proteome profiling to monitor health and disease. *Nat Commun* **12**, 2493, doi:10.1038/s41467-021-22767-z (2021).

5 Zhong, W. *et al.* Whole-genome sequence association analysis of blood proteins in a longitudinal wellness cohort. *Genome Med* **12**, 53, doi:10.1186/s13073-020-00755-0 (2020).

6 Liao, Y., Wang, J., Jaehnig, E. J., Shi, Z. & Zhang, B. WebGestalt 2019: gene set analysis toolkit with revamped UIs and APIs. *Nucleic Acids Res* **47**, W199-W205, doi:10.1093/nar/gkz401 (2019).

7 Rezatabar, S. *et al.* RAS/MAPK signaling functions in oxidative stress, DNA damage response and cancer progression. *J Cell Physiol* **234**, 14951-14965, doi:10.1002/jcp.28334 (2019).

8 Shang, F. & Taylor, A. Ubiquitin-proteasome pathway and cellular responses to oxidative stress. *Free Radic Biol Med* **51**, 5-16, doi:10.1016/j.freeradbiomed.2011.03.031 (2011).

9 Zaghlool, S. B. *et al.* Revealing the role of the human blood plasma proteome in obesity using genetic drivers. *Nat Commun* **12**, 1279, doi:10.1038/s41467-021-21542-4 (2021).

10 Tanaka, T. *et al.* Plasma proteomic biomarker signature of age predicts health and life span. *Elife* **9**, doi:10.7554/eLife.61073 (2020).

11 Sun, B. B. *et al.* Genomic atlas of the human plasma proteome. *Nature* **558**, 73-79, doi:10.1038/s41586-018-0175-2 (2018).

12 Ngo, D. *et al.* Aptamer-Based Proteomic Profiling Reveals Novel Candidate Biomarkers and Pathways in Cardiovascular Disease. *Circulation* **134**, 270-285, doi:10.1161/CIRCULATIONAHA.116.021803 (2016).

13 Menni, C. *et al.* Circulating Proteomic Signatures of Chronological Age. *J Gerontol A Biol Sci Med Sci* **70**, 809-816, doi:10.1093/gerona/glu121 (2015).

14 Ferkingstad, E. *et al.* Large-scale integration of the plasma proteome with genetics and disease. *Nat Genet* **53**, 1712-1721, doi:10.1038/s41588-021-00978-w (2021).

15 Katz, D. H. *et al.* Proteomic profiling platforms head to head: Leveraging genetics and clinical traits to compare aptamer- and antibody-based methods. *Sci Adv* **8**, eabm5164, doi:10.1126/sciadv.abm5164 (2022).

16 Burger, H. G., Hale, G. E., Robertson, D. M. & Dennerstein, L. A review of hormonal changes during the menopausal transition: focus on findings from the Melbourne Women's Midlife Health Project. *Hum Reprod Update* **13**, 559-565, doi:10.1093/humupd/dmm020 (2007).

17 Uchida, H. *et al.* Glycodelin in reproduction. *Reprod Med Biol* **12**, 79-84, doi:10.1007/s12522-013-0144-2 (2013).
18 Wang, D. *et al.* GDF15: emerging biology and therapeutic applications for obesity and cardiometabolic disease. *Nat Rev Endocrinol* **17**, 592-607, doi:10.1038/s41574-021-00529-7 (2021).
19 Burkhardt, A. M. *et al.* CXCL17 is a major chemotactic factor for lung macrophages. *J Immunol* **193**, 1468-1474, doi:10.4049/jimmunol.1400551 (2014).
20 Inker, L. A. *et al.* Estimating glomerular filtration rate from serum creatinine and cystatin C. *N Engl J Med* **367**, 20-29, doi:10.1056/NEJMoa1114248 (2012).
21 Niewczas, M. A. *et al.* A signature of circulating inflammatory proteins and development of end-stage renal disease in diabetes. *Nat Med* **25**, 805-813, doi:10.1038/s41591-019-0415-5 (2019).
22 Ku, N. O., Strnad, P., Bantel, H. & Omary, M. B. Keratins: Biomarkers and modulators of apoptotic and necrotic cell death in the liver. *Hepatology* **64**, 966-976, doi:10.1002/hep.28493 (2016).
23 Gudjonsson, A. *et al.* A genome-wide association study of serum proteins reveals shared loci with common diseases. *Nat Commun* **13**, 480, doi:10.1038/s41467-021-27850-z (2022).
24 Folkersen, L. *et al.* Genomic and drug target evaluation of 90 cardiovascular proteins in 30,931 individuals. *Nat Metab* **2**, 1135-1148, doi:10.1038/s42255-020-00287-2 (2020).
25 Asimit, J. L. *et al.* Stochastic search and joint fine-mapping increases accuracy and identifies previously unreported associations in immune-mediated diseases. *Nat Commun* **10**, 3216, doi:10.1038/s41467-019-11271-0 (2019).
26 Benner, C. *et al.* FINEMAP: efficient variable selection using summary data from genome-wide association studies. *Bioinformatics* **32**, 1493-1501, doi:10.1093/bioinformatics/btw018 (2016).
27 Wang, G., Sarkar, A., Carbonetto, P. & Stephens, M. A simple new approach to variable selection in regression, with application to genetic fine mapping. *Journal of the Royal Statistical Society: Series B (Statistical Methodology)* **82**, 1273-1300, doi:https://doi.org/10.1111/rssb.12388 (2020).
28 Yang, J. *et al.* Conditional and joint multiple-SNP analysis of GWAS summary statistics identifies additional variants influencing complex traits. *Nat Genet* **44**, 369-375, S361-363, doi:10.1038/ng.2213 (2012).
29 Pietzner, M. *et al.* Genetic architecture of host proteins involved in SARS-CoV-2 infection. *Nat Commun* **11**, 6397, doi:10.1038/s41467-020-19996-z (2020).
30 Astle, W. J. *et al.* The Allelic Landscape of Human Blood Cell Trait Variation and Links to Common Complex Disease. *Cell* **167**, 1415-1429 e1419, doi:10.1016/j.cell.2016.10.042 (2016).
31 Uhlen, M. *et al.* A genome-wide transcriptomic analysis of protein-coding genes in human blood cells. *Science* **366**, doi:10.1126/science.aax9198 (2019).
32 Labayen, I. *et al.* Association between the FTO rs9939609 polymorphism and leptin in European adolescents: a possible link with energy balance control. The HELENA study. *Int J Obes (Lond)* **35**, 66-71, doi:10.1038/ijo.2010.219 (2011).
33 Kelly, R. J., Rouquier, S., Giorgi, D., Lennon, G. G. & Lowe, J. B. Sequence and expression of a candidate for the human Secretor blood group alpha(1,2)fucosyltransferase gene (FUT2). Homozygosity for an enzyme-inactivating

nonsense mutation commonly correlates with the non-secretor phenotype. *J Biol Chem* **270**, 4640-4649, doi:10.1074/jbc.270.9.4640 (1995).
34 Chiou, J. *et al.* Interpreting type 1 diabetes risk with genetics and single-cell epigenomics. *Nature* **594**, 398-402, doi:10.1038/s41586-021-03552-w (2021).
35 Donertas, H. M., Fabian, D. K., Valenzuela, M. F., Partridge, L. & Thornton, J. M. Common genetic associations between age-related diseases. *Nat Aging* **1**, 400-412, doi:10.1038/s43587-021-00051-5 (2021).
36 de Lange, K. M. *et al.* Genome-wide association study implicates immune activation of multiple integrin genes in inflammatory bowel disease. *Nat Genet* **49**, 256-261, doi:10.1038/ng.3760 (2017).
37 Masuda, M., Okuda, K., Ikeda, D. D., Hishigaki, H. & Fujiwara, T. Interaction of genetic markers associated with serum alkaline phosphatase levels in the Japanese population. *Hum Genome Var* **2**, 15019, doi:10.1038/hgv.2015.19 (2015).
38 Kim, Y. S. & Ho, S. B. Intestinal goblet cells and mucins in health and disease: recent insights and progress. *Curr Gastroenterol Rep* **12**, 319-330, doi:10.1007/s11894-010-0131-2 (2010).
39 Tong, M. *et al.* Reprograming of gut microbiome energy metabolism by the FUT2 Crohn's disease risk polymorphism. *ISME J* **8**, 2193-2206, doi:10.1038/ismej.2014.64 (2014).
40 Ye, B. D. *et al.* Association of FUT2 and ABO with Crohn's disease in Koreans. *J Gastroenterol Hepatol* **35**, 104-109, doi:10.1111/jgh.14766 (2020).
41 Liang, W. *et al.* FAM3D is essential for colon homeostasis and host defense against inflammation associated carcinogenesis. *Nat Commun* **11**, 5912, doi:10.1038/s41467-020-19691-z (2020).
42 Karlsson, M. *et al.* A single-cell type transcriptomics map of human tissues. *Sci Adv* **7**, doi:10.1126/sciadv.abh2169 (2021).
43 Parlato, M. *et al.* Human ALPI deficiency causes inflammatory bowel disease and highlights a key mechanism of gut homeostasis. *EMBO Mol Med* **10**, doi:10.15252/emmm.201708483 (2018).
44 Forni, D. *et al.* ABO histo-blood group might modulate predisposition to Crohn's disease and affect disease behavior. *J Crohns Colitis* **8**, 489-494, doi:10.1016/j.crohns.2013.10.014 (2014).
45 Uhlen, M. *et al.* Proteomics. Tissue-based map of the human proteome. *Science* **347**, 1260419, doi:10.1126/science.1260419 (2015).
46 Shen, Y. *et al.* A map of the cis-regulatory sequences in the mouse genome. *Nature* **488**, 116-120, doi:10.1038/nature11243 (2012).
47 Foley, C. N. *et al.* A fast and efficient colocalization algorithm for identifying shared genetic risk factors across multiple traits. *Nat Commun* **12**, 764, doi:10.1038/s41467-020-20885-8 (2021).
48 Nakano, Y. *et al.* MUC5B Promoter Variant rs35705950 Affects MUC5B Expression in the Distal Airways in Idiopathic Pulmonary Fibrosis. *Am J Respir Crit Care Med* **193**, 464-466, doi:10.1164/rccm.201509-1872LE (2016).
49 Obeidat, M. *et al.* Surfactant protein D is a causal risk factor for COPD: results of Mendelian randomisation. *Eur Respir J* **50**, doi:10.1183/13993003.00657-2017 (2017).

50 Palmos, A. B. *et al.* Proteome-wide Mendelian randomization identifies causal links between blood proteins and severe COVID-19. *PLoS Genet* **18**, e1010042, doi:10.1371/journal.pgen.1010042 (2022).
51 Islam, A. & Khan, M. A. Lung transcriptome of a COVID-19 patient and systems biology predictions suggest impaired surfactant production which may be druggable by surfactant therapy. *Sci Rep* **10**, 19395, doi:10.1038/s41598-020-76404-8 (2020).
52 Olajuyin, A. M., Zhang, X. & Ji, H. L. Alveolar type 2 progenitor cells for lung injury repair. *Cell Death Discov* **5**, 63, doi:10.1038/s41420-019-0147-9 (2019).
53 Huang, J. *et al.* SARS-CoV-2 Infection of Pluripotent Stem Cell-Derived Human Lung Alveolar Type 2 Cells Elicits a Rapid Epithelial-Intrinsic Inflammatory Response. *Cell Stem Cell* **27**, 962-973 e967, doi:10.1016/j.stem.2020.09.013 (2020).
54 Ramana, C. V. & Das, B. Regulation of Lysosomal Associated Membrane Protein 3 (LAMP3) in Lung Epithelial Cells by Coronaviruses (SARS-CoV-1/2) and Type I Interferon Signaling. *Computational and Mathematical Biophysics* **10**, 167-183, doi:doi:10.1515/cmb-2022-0140 (2022).
55 van de Vosse, E. *et al.* IL-12Rbeta1 deficiency: mutation update and description of the IL12RB1 variation database. *Hum Mutat* **34**, 1329-1339, doi:10.1002/humu.22380 (2013).
56 Broz, P. & Dixit, V. M. Inflammasomes: mechanism of assembly, regulation and signalling. *Nat Rev Immunol* **16**, 407-420, doi:10.1038/nri.2016.58 (2016).
57 Lamkanfi, M. & Dixit, V. M. Mechanisms and functions of inflammasomes. *Cell* **157**, 1013-1022, doi:10.1016/j.cell.2014.04.007 (2014).
58 Welzel, T. & Kuemmerle-Deschner, J. B. Diagnosis and Management of the Cryopyrin-Associated Periodic Syndromes (CAPS): What Do We Know Today? *J Clin Med* **10**, doi:10.3390/jcm10010128 (2021).
59 Malcova, H. *et al.* IL-1 Inhibitors in the Treatment of Monogenic Periodic Fever Syndromes: From the Past to the Future Perspectives. *Front Immunol* **11**, 619257, doi:10.3389/fimmu.2020.619257 (2020).
60 Pietzner, M. *et al.* Cross-platform proteomics to advance genetic prioritisation strategies. *bioRxiv*, 2021.2003.2018.435919, doi:10.1101/2021.03.18.435919 (2021).
61 Meissner, T. B. *et al.* NLR family member NLRC5 is a transcriptional regulator of MHC class I genes. *Proc Natl Acad Sci U S A* **107**, 13794-13799, doi:10.1073/pnas.1008684107 (2010).
62 Dang, A. T. *et al.* NLRC5 promotes transcription of BTN3A1-3 genes and Vgamma9Vdelta2 T cell-mediated killing. *iScience* **24**, 101900, doi:10.1016/j.isci.2020.101900 (2021).
63 Joehanes, R. *et al.* Integrated genome-wide analysis of expression quantitative trait loci aids interpretation of genomic association studies. *Genome Biol* **18**, 16, doi:10.1186/s13059-016-1142-6 (2017).
64 Motyan, J. A., Bagossi, P., Benko, S. & Tozser, J. A molecular model of the full-length human NOD-like receptor family CARD domain containing 5 (NLRC5) protein. *BMC Bioinformatics* **14**, 275, doi:10.1186/1471-2105-14-275 (2013).
65 Kobayashi, K. S. & van den Elsen, P. J. NLRC5: a key regulator of MHC class I-dependent immune responses. *Nat Rev Immunol* **12**, 813-820, doi:10.1038/nri3339 (2012).
66 Giugliano, R. P. *et al.* Stroke Prevention With the PCSK9 (Proprotein Convertase Subtilisin-Kexin Type 9) Inhibitor Evolocumab Added to Statin in High-Risk Patients

With Stable Atherosclerosis. *Stroke* **51**, 1546-1554, doi:10.1161/STROKEAHA.119.027759 (2020).
67 Karatasakis, A. *et al.* Effect of PCSK9 Inhibitors on Clinical Outcomes in Patients With Hypercholesterolemia: A Meta-Analysis of 35 Randomized Controlled Trials. *J Am Heart Assoc* **6**, doi:10.1161/JAHA.117.006910 (2017).
68 Sabatine, M. S. *et al.* Efficacy and safety of evolocumab in reducing lipids and cardiovascular events. *N Engl J Med* **372**, 1500-1509, doi:10.1056/NEJMoa1500858 (2015).
69 Robinson, J. G. *et al.* Efficacy and safety of alirocumab in reducing lipids and cardiovascular events. *N Engl J Med* **372**, 1489-1499, doi:10.1056/NEJMoa1501031 (2015).
70 Pott, J. *et al.* Meta-GWAS of PCSK9 levels detects two novel loci at APOB and TM6SF2. *Hum Mol Genet* **31**, 999-1011, doi:10.1093/hmg/ddab279 (2022).
71 Macdonald-Dunlop, E. *et al.* Mapping genetic determinants of 184 circulating proteins in 26,494 individuals to connect proteins and diseases. *medRxiv*, 2021.2008.2003.21261494, doi:10.1101/2021.08.03.21261494 (2021).
72 Filippatos, T. D., Kei, A., Rizos, C. V. & Elisaf, M. S. Effects of PCSK9 Inhibitors on Other than Low-Density Lipoprotein Cholesterol Lipid Variables. *J Cardiovasc Pharmacol Ther* **23**, 3-12, doi:10.1177/1074248417724868 (2018).
73 Pietzner, M. *et al.* Mapping the proteo-genomic convergence of human diseases. *Science* **374**, eabj1541, doi:10.1126/science.abj1541 (2021).
74 Gilly, A. *et al.* Whole-genome sequencing analysis of the cardiometabolic proteome. *Nat Commun* **11**, 6336, doi:10.1038/s41467-020-20079-2 (2020).
75 Suhre, K. *et al.* Connecting genetic risk to disease end points through the human blood plasma proteome. *Nat Commun* **8**, 14357, doi:10.1038/ncomms14357 (2017).
