# Supplementary material for: Plasma proteomic associations with genetics and health in the UK Biobank
Source: Nature. 2023 Oct 4;622(7982):329–38. doi: 10.1038/s41586-023-06592-6 (PMC10567551; doi:10.1038/s41586-023-06592-6)
Supplement: Supplementary file 2 — Reporting Summary [file 41586_2023_6592_MOESM2_ESM.pdf]

nature portfolio

Corresponding author(s): Christopher D. Whelan
Benjamin B. Sun

Last updated by author(s): Jul 31, 2023

Reporting Summary

Nature Portfolio wishes to improve the reproducibility of the work that we publish. This form provides structure for consistency and transparency in reporting. For further information on Nature Portfolio policies, see our Editorial Policies and the Editorial Policy Checklist.

Statistics

For all statistical analyses, confirm that the following items are present in the figure legend, table legend, main text, or Methods section.

n/a	Confirmed	
☐	☒	The exact sample size (*n*) for each experimental group/condition, given as a discrete number and unit of measurement
☐	☒	A statement on whether measurements were taken from distinct samples or whether the same sample was measured repeatedly
☐	☒	The statistical test(s) used AND whether they are one- or two-sided<br>*Only common tests should be described solely by name; describe more complex techniques in the Methods section.*
☐	☒	A description of all covariates tested
☐	☒	A description of any assumptions or corrections, such as tests of normality and adjustment for multiple comparisons
☐	☒	A full description of the statistical parameters including central tendency (e.g. means) or other basic estimates (e.g. regression coefficient) AND variation (e.g. standard deviation) or associated estimates of uncertainty (e.g. confidence intervals)
☐	☒	For null hypothesis testing, the test statistic (e.g. *F*, *t*, *r*) with confidence intervals, effect sizes, degrees of freedom and *P* value noted<br>*Give P values as exact values whenever suitable.*
☐	☒	For Bayesian analysis, information on the choice of priors and Markov chain Monte Carlo settings
☒	☐	For hierarchical and complex designs, identification of the appropriate level for tests and full reporting of outcomes
☐	☒	Estimates of effect sizes (e.g. Cohen's *d*, Pearson's *r*), indicating how they were calculated

Our web collection on statistics for biologists contains articles on many of the points above.

Software and code

Policy information about availability of computer code

Data collection	R (v3.6.1)
Data analysis	R (v3.6.1)<br>REGENIE v2.2.1<br>PLINK v1.9 and v2.0<br>VEP v105<br>ANNOVAR v20211019<br>Ingenuity Pathway Analysis (QIAGEN IPA)<br>WGSA v0.95<br>SuSIE v0.12.6<br>HyPrColoc R package v1.0<br>TissueEnrich R package v1.6.0<br>LiftOver R package 1.20<br>TwoSampleMR R package v0.5.6<br>mrpipeline R package v0.1<br>ieugwasr R package v0.1.5<br>biomaRt R package v2.52.0<br>gwasvcf R package v0.1.0<br>coloc R package v5.1.0

For manuscripts utilizing custom algorithms or software that are central to the research but not yet described in published literature, software must be made available to editors and reviewers. We strongly encourage code deposition in a community repository (e.g. GitHub). See the Nature Portfolio guidelines for submitting code & software for further information.

Data

Policy information about availability of data

All manuscripts must include a data availability statement. This statement should provide the following information, where applicable:

- Accession codes, unique identifiers, or web links for publicly available datasets
- A description of any restrictions on data availability
- For clinical datasets or third party data, please ensure that the statement adheres to our policy

his is included in the Main Text. An interactive portal for the proteo-genomic results and summary association data are available at https://ukb-ppp.azurewebsites.net/. Underlying proteomics data is available through the UK Biobank Research Access Portal.

Field-specific reporting

Please select the one below that is the best fit for your research. If you are not sure, read the appropriate sections before making your selection.

☒ Life sciences ☐ Behavioural & social sciences ☐ Ecological, evolutionary & environmental sciences

For a reference copy of the document with all sections, see nature.com/documents/nr-reporting-summary-flat.pdf

Life sciences study design

All studies must disclose on these points even when the disclosure is negative.

Sample size	54,219 participants; sample size was not predetermined as lower bound of genetic and non-genetic is unknown.
Data exclusions	Exclusions as part of QC have been detailed in Supplementary Information and Methods
Replication	Internal replication cohort (reported in results) and external replication in independent studies as detailed in the Results and Supplementary Information
Randomization	NA - non-interventional
Blinding	NA - non-interventional

Reporting for specific materials, systems and methods

We require information from authors about some types of materials, experimental systems and methods used in many studies. Here, indicate whether each material, system or method listed is relevant to your study. If you are not sure if a list item applies to your research, read the appropriate section before selecting a response.

Materials & experimental systems

n/a	Involved in the study	
☐	☒	Antibodies
☒	☐	Eukaryotic cell lines
☒	☐	Palaeontology and archaeology
☒	☐	Animals and other organisms
☐	☒	Human research participants
☒	☐	Clinical data
☒	☐	Dual use research of concern

Methods

n/a	Involved in the study	
☒	☐	ChIP-seq
☒	☐	Flow cytometry
☒	☐	MRI-based neuroimaging

Antibodies

Antibodies used	A full list of proteins measured using the antibody-based Olink Explore 3072 is provided on the Olink website: https://olink.com/products-services/explore/ All assays in Olink's panels use antigen affinity-purified polyclonal or monoclonal antibodies (or combinations of both), with the majority being commercially available.
Validation	Validation data for the Explore 3072 assay are available on the Olink website: https://olink.com/products-services/explore/

Human research participants

Policy information about studies involving human research participants

Population characteristics	UK Biobank comprises up to 502,650 participants aged between 40 to 69 years at baseline recruited across 22 assessment centres in England, Scotland and Wales. The average age at baseline was 56.52 years (standard deviation, SD 8.09). Of the 502,650 volunteers, 273,468 were women (54.41%), who were on average younger than the men (56.35 years, SD 8.00). Additional details are provided in Hewitt et al. (BMJ Open, 2016).<br><br>A comparison of the UK Biobank with individuals in the general population, conducted by Fry et al. (American Journal of Epidemiology, 2017) found that UKB participants were, "more likely to be older, to be female, and to live in less socioeconomically deprived areas than nonparticipants", suggesting evidence of a selection bias towards healthy volunteers.
Recruitment	The recruitment strategy for UK Biobank is described in detail by Bycroft et al (Nature, 2018). Briefly, participants aged 40 to 69 years were recruited across the United Kingdom between the years 2006 and 2010 from the National Health Service (NHS) patient registers. Approximately 9.2 million people living 25 miles (40 km) from one of 22 assessment centers across England, Wales and Scotland were invited to participate, with 5.5% participating in the baseline studies. All participants completed self-report questionnaires detailing their demographic, socioeconomic and health-related characteristics. Participants also underwent several physical assessments (e.g., repeated blood pressure measurements, weight and height). Participants also provided blood, urine and saliva samples, which were then stored in a central storage facility in Stockport, United Kingdom.
Ethics oversight	Ethics approval for the UK Biobank study was obtained from the North West Centre for Research Ethics Committee (11/NW/0382). Proteomic profiling of the UK Biobank was approved by the Access Subcommittee of UK Biobank, under Access Management System Application No. 65851.

Note that full information on the approval of the study protocol must also be provided in the manuscript.
